# Macrophage polarization regulated by the βTrCP-NF-κB inflammatory signaling pathway is required for valproic acid’s dual role in breast tumor radiosensitivity and normal tissue radioprotection

**DOI:** 10.3389/fimmu.2026.1839063

**Published:** 2026-08-10

**Authors:** Beidi Jia, Guochao Liu, David Lim, Quanji Li, Songying Xie, Zhihui Feng

**Affiliations:** 1 Department of Occupational and Environmental Health, School of Public Health, Cheeloo College of Medicine, Shandong University, Shandong, Jinan, China; 2 Faculty of Health, University of Technology Sydney, Ultimo, NSW, Australia

**Keywords:** breast tumor, macrophages, NF-κB, radiation, VPA, βTrCP

## Abstract

Radiotherapy (RT) is a widely used treatment modality for breast cancer, but its broad application is limited by the risk of adverse effects, such as the potential for the tumor to develop resistance to RT. Identifying strategies that enhance RT efficacy while minimizing adverse effects is of significant clinical importance. Valproic acid (VPA), an anti-epileptic drug, has shown promise in augmenting the efficacy of RT, though its underlying mechanisms remain unclear. We established *in vitro* co-culture systems and in vivo breast tumor models, and performed multiple molecular and cellular assays to investigate the immunological mechanism underlying VPA-mediated tumor radiosensitization. In this study, we demonstrated that VPA enhances the radiosensitivity of breast tumor by activating the βTrCP-NF-κB immune pathway, which reprograms tumor-associated macrophages (TAMs) into the M1 phenotype. VPA suppresses HDAC1/2 activity, which in turn upregulates βTrCP expression, accelerating ubiquitination and degradation of the NF-κB suppressor IκBα. In normal tissues, VPA suppresses the inflammatory response triggered by the βTrCP-NF-κB pathway. This bidirectional regulatory effect of VPA reveals a novel immunological mechanism for its application as a RT adjuvant, offering both enhanced tumor radiosensitivity and radioprotection of normal tissues.

## Introduction

Breast cancer is one of the most common types of cancer globally, with approximately 2.3 million new cases reported worldwide each year, accounting for 11.6% of all cancer diagnoses (1). Breast cancer remains the leading cause of cancer-related death among women, making its prevention and treatment a major global health priority. Radiotherapy (RT) is a mainstay of breast cancer management due to its availability, broad application and patient tolerance (2). However, RT’s efficacy may be limited by individual variability in radiosensitivity, the potential for tumors to develop radioresistance and tumor recurrence (3, 4). Furthermore, while ionizing radiation (IR) directly kills tumor tissue, it can also damage surrounding normal tissues, leading to radiation abscopal effect (RAE) and significantly limiting therapeutic outcomes. There is a pressing need for new treatment strategies that can increase radiosensitivity while protecting normal tissues, ultimately improving patient survival and prognosis (5).

Histone deacetylase inhibitors (HDACis), epigenetic factors, have emerged as a novel anti-cancer therapeutic (6, 7). Among them, valproic acid (VPA), a class I and IIa HDACi, conventionally used for epilepsy, has shown much promise (8). Previous studies have shown that VPA acts as a multi-target radiosensitizer, inhibiting DNA damage repair and activating immune responses in tumor tissues (9, 10). Furthermore, VPA has been shown to alleviate IR-damage to rodent brain cells, hair loss in patients with glioma and improve survival (11, 12). Collectively, these findings suggest that VPA may serve as a potential adjuvant for RT and as a radioprotector of non-targeted normal tissues. However, the immune mechanisms underlying this bidirectional effect remain unclear.

IR stimulates the release of pro-inflammatory mediators and chemokines by tumor and stromal cells, promotes the infiltration of immune cells, and activates the tumor immune microenvironment (TIME). Changes in TIME induced by RT are closely linked to tumor progression (13). Macrophages are key immunologically active cells in the irradiated tumor microenvironment (ITME) (14). Tumor-associated macrophages (TAMs) are classified into immunosuppressive M2 phenotype and immunostimulatory M1 phenotype. Cytokines such as M-CSF/CSF1, IL-4/IL-13, IL-6, IL-10, and TGF-β drive M2 TAMs polarization, while GM-CSF/CSF2, TNF-α, and IFN-γ promote M1 TAMs polarization (15). As tumors progress, the immunosuppressive M2 TAMs increase, suppressing anti-tumor immunity, and contributing to a “cold” TIME that favors tumor growth (16, 17). RT alone is often insufficient to shift the balance towards the anti-tumor effect mediated by M1 TAMs (14), highlighting the need for combination therapies that can effectively reprogram TAMs. In addition, the death of tumor cells induced by IR can release tumor-associated antigens and damage-associated molecular patterns, thereby affecting TIME and promote the occurrence of radiation-induced RAE (18–20). Macrophages are known to mediate RAE by releasing large amounts of inflammatory factors and activating signaling pathways, such as ROS, NO, and MAPKs (21, 22). It is evident that macrophages play a crucial regulatory role in tumor radioresistance and RAE.

Nuclear factor kappa B (NF-κB) plays a central role in the development and progression of tumor, as well as in inflammation and immune modulation in the TIME (23). IR activates NF-κB via phosphorylation and subsequent ubiquitination and degradation of NF-κB inhibitor alpha (IκBα), leading to the release of the NF-κB dimer and the nuclear translocation of p65, and transcription of pro-inflammatory genes, such as TNF-α, IL-6, IL-1α and IL-1β, recruiting immune cells to the TIME, and enhancing the inflammatory response of the tumor (14, 24–26). Impaired NF-κB activation reduces RT efficacy (27). NF-κB also regulates macrophage development and polarization (28), and VPA has been reported to affect NF-κB phosphorylation in tumor cells (29). However, it remains unclear whether VPA modulates NF-κB signaling to enhance tumor radio-sensitivity and inhibit RAE.

In fact, the NF-κB complex is confined to the cytoplasm through interaction with the IκB protein family, thereby maintaining its inactive state. After stimulations, the IκB protein is phosphorylated at the serine 32 and 36 sites by the IκB kinase (IKK) complex (30–32). Subsequently, the phosphorylated IκBα is ubiquitinated by SKP1-CUL1-F-box (SCF) E3 ubiquitin ligase complex and degraded rapidly (33). β-transducin repeat-containing protein (βTrCP) acts as a subunit of the host SCF E3 ubiquitin protein ligase complex, which can recognize phosphorylated IκBα and rapidly mediate its ubiquitination and degradation, thereby inducing NF-κB to enter the cell nucleus (34). Studies found that upregulation of βTrCP accelerated the degradation of IκBα, thereby enhancing NF-κB activity (35). βTrCP plays a crucial role in the activation of the NF-κB signaling pathway (36) and affects breast cancer progression (37). As the key E3 ligase regulating the ubiquitination degradation of NF-κB/IκBα, functional analysis indicated that βTrCP may be associated with TAMs polarization and the subsequent inflammatory responses (38), but the role of βTrCP-NF-κB axis in the ITME and its regulation by HDACis are not well understood. We hypothesized that VPA enhances breast cancer radiosensitivity and prevents RAE by modulating TAMs polarization through the βTrCP-NF-κB inflammatory response pathway.

## Methods and materials

### Establishment of the breast cancer animal models

Female BALB/c mice (7 weeks old, 18–20 g) were purchased from Beijing Vital River Laboratory Animal Technology Co., Ltd., Beijing, China. The female Sprague-Dawley (SD) rats (45 days old, 120g ± 5 g) were purchased from Pengyue Laboratory Animal Co., Ltd., Jinan, China. The studies of animal tissue were performed in accordance with the requirements of the Shandong University Human and Animal Ethics Research Committee (LL202303041). All the experimental animals were placed in an environment free of specific pathogens, with the temperature maintained at 23±1 °C and the relative humidity maintained at 30-70%. The light cycle was 12 h per day, and during the experiment, the animals were allowed free access to irradiated food and water. The care of these animals complies with the relevant laws and guidelines concerning experiments and scientific uses in China. The mice were randomly divided into the 4T1 group, the 4T1+VPA (VPA, Cat. No. P4543, Sigma-Aldrich, USA) group, the 4T1+IR group, and the 4T1+IR+VPA group. And the rats were randomly divided into the DMBA (Cat. No. D3254, Sigma-Aldrich, USA) group, the DMBA+VPA group, the DMBA+IR group, and the DMBA+IR+VPA group.

The concentration of 4T1-Luc cells (a gift from Dr Xiuli Guo at Shandong University) in a healthy growth state was adjusted to 2×10^5^ cells per 100 µl using phosphate-buffered saline (PBS; Cat. No. G4202, Servicebio, China). The 100 µl cell suspension was subcutaneously inoculated into the lower right fourth pair of mammary fat pads of 8-week-old BALB/c mice. The formation of breast tumors was confirmed based on morphological observation, hematoxylin and eosin (HE) staining, and bioluminescence imaging. The detailed establishment method of the primary SD rat breast tumor animal model induced by DMBA has been described elsewhere (9, 10). The tumor size was measured with calipers and calculated as (L × W^2^)/2, where L and W are the longest and shortest diameters of the tumor, respectively, in millimeters (mm).

### Treatments of tumor-bearing mice

The administration of IR and VPA was determined based on equivalent clinical treatment doses. The VPA dose for the animals was 200 mg/kg, administered twice daily for 6 consecutive days (intraperitoneal injection, *i.p.*). The collimator was utilized to achieve precise irradiation of the breast tumor area using a fractionated irradiation of 2 Gy per fraction for 4 consecutive fractions using an X-ray Irradiator (X-RAD225 OptiMAX, Pxi, USA). The detailed method was described elsewhere (9, 10).

### Establishment of the macrophage depletion model in 4T1-induced mouse breast tumors

We used clodronate liposomes (CL; Cat. No. C-010, LIPOSOMA, Netherlands) to establish a macrophage-depleting model in the 4T1-induced mouse breast xenograft model. The control group received PBS (Cat. No. G4202, Servicebio, China). When the average tumor volume reached 100 mm^3^, mice were treated with CL (100 µl/10 g, *i.p.*) every three days and simultaneously received VPA and IR.

### Inhibitor treatments in the mouse breast transplant tumor model

We used QNZ (Cat. No. S4902, Selleck Chemicals, USA) and GS143 (Cat. No. HY-110261, MedChemExpress, USA) to inhibit the activation of the NF-κB and βTrCP signaling pathways in the 4T1 mouse breast tumor model, respectively. The control group received saline. The dose of QNZ was 0.4 mg/kg (once a day, *i.p.*), while the dose of GS143 was 2 mg/kg (once a day, *i.p.*) until the end of the experiment.

### Bioluminescence imaging *in vivo*

The mice were treated with D-Luciferin potassium (Cat. No. MB1834, Meilunbio, China) at a concentration of 15 mg/ml before being anesthetized and the dose was 10 µl/g (*i.p.*). Observation was performed using a Small Animal *In Vivo* Optical Imaging System (IVIS Spectrum, PerkinElmer, USA) after the animal was anesthetized.

### BrdU incorporation and HE staining

5-Bromo-2’-deoxyuridine (BrdU, Cat. No. B5002, Sigma-Aldrich, USA) at a dose of 100 mg/kg was injected *i.p.* 24 h before tissue harvest. The animal tissues were fixed in a 4% paraformaldehyde solution overnight, then embedded in paraffin. They were sectioned continuously at 5 μm according to the manufacturer’s instructions and stained with HE. The paraffin-embedded tissue sections were deparaffinized in xylene, hydrated with different concentrations of ethanol, stained with hematoxylin (Cat. No. G1004, Servicebio, China) for 2 min to reveal cell nuclei and stained with eosin (Cat. No. G1002, Servicebio, China) for 1 min to color the cytoplasmic components. The sections were then dehydrated, cleared in xylene, and mounted for microscopic examination (Olympus Corporation of Japan).

### Immunohistochemistry

The tissue sections were first deparaffinized in xylene and hydrated using a gradient of 100% to 75% ethanol. Then the sections were boiled in a sodium citrate antigen retrieval solution at temperatures above 92 °C for 20 min. For the 5-bromo-2’-deoxyuridine staining, the sections were incubated in 1 M hydrochloric acid for 1 h, then neutralized in 0.1 M sodium borate buffer for 10 min. Next, the sections were incubated in 3% hydrogen peroxide for 15 min to block endogenous peroxidase, then incubated with goat serum for 1 h. Subsequently, these sections were incubated with the primary antibodies overnight at 4 °C. The primary antibodies used were: BrdU (1:50; Cat. No. 347580, BD Biosciences, USA), Ki67 (1:400; Cat. No. 12202, Cell Signaling Technology, USA), F4/80 (1:5000; Cat. No. ab300421, Abcam, UK), CD80 (1:100; Cat. No. sc-376012; Santa Cruz Biotechnology, USA), CD206 (1:200; Cat. No. 24595; Cell Signaling Technology, USA), CD68 (1:800, Cat. No. GB11067, Servicebio, China), CD11b (1:5000, Cat. No. ab133357, Abcam, UK). The primary antibodies were diluted with Tris-Buffered Saline (TBS; Cat. No. G0001, Servicebio, China) with Tween-20 (Cat. No. T8220, Solarbio, China) and 1% bovine serum albumin (BSA, Cat. No. 9048-46-8, Yeasen, China). Sections were subsequently incubated with secondary antibodies: biotinylated goat anti-rabbit IgG (1:300, Cat. No. BA-1000, Vector Laboratories, USA), biotinylated goat anti-mouse IgG (1:300, Cat. No. BA-9200, Vector Laboratories, USA), biotinylated goat anti-rat IgG (1:300, Cat. No. BA-9400, Vector Laboratories, USA) for 1 h at room temperature. After incubation with the avidin-biotin complex (Cat. No. PK-4000, Vector Laboratories, USA) in conjunction with 3,3’-diaminobenzidine (DAB, Cat. No. ZLI-9018, ZSGB-Bio, China) staining, hematoxylin and counterstaining were followed. The sections were dehydrated and mounted for observation. Images were taken through a light microscope (Olympus Corporation of Japan).

### TUNEL staining

One-step TUNEL FITC Apoptosis Detection Kit (Cat. No. K1133, APE×BIO, USA) was used to detect mouse tumor tissue sections according to the standard protocol. After the section was deparaffinized and hydrated, it was incubated with 100 µL of proteinase K (Cat. No. K1133, APE×BIO, USA) working solution, then washed with 1× PBS. Next, the sections were incubated with 1× equilibrium buffer working solution at 37 °C for 30 min, then incubated with labeled reaction solution at 37 °C for 1 h in the dark. The labeled reaction solution was prepared according to the instructions. After the sections were washed with PBS, they were stained with DAPI (4’,6-diamidino-2-phenylindole) solution (Cat. No. G1012, Servicebio, China) and sealed with the anti-fluorescence quenching agent (Cat. No. G1401, Servicebio, China). The sections were observed under a fluorescence microscope (Olympus Corporation of Japan), green fluorescence was observed at 517 nanometers (nm), and blue fluorescence of DAPI was observed at 460 nm.

### Western blotting and cell immunoprecipitation (for detecting protein ubiquitination)

The protein samples were extracted using RIPA lysis buffer (Cat. No. G2002, Servicebio, China) containing protease inhibitor (Cat. No. K1007, APE×BIO, USA) and phosphatase inhibitor (Cat. No. G2007, Servicebio, China). After the protein samples were centrifuged, they were quantified using Pierce™ Microplate BCA Protein Assay Kit (Cat. No. 23252, Thermo Fisher Scientific, USA). The protocol is as follows: SDS-PAGE gel making (Cat. No. G2042, G2043, 2044, Servicebio, China), sample loading, electrophoresis, transferring to polyvinylidene fluoride (PVDF) membranes (Cat. No. 36125ES03, Yeasen, China), milk blocking, incubation with primary and secondary antibodies, and chemiluminescence. The primary antibodies used were: p65 (1:1000; Cat. No. 8242, Cell Signaling Technology, USA), p-p65 (1:1000; Cat. No. 3033, Cell Signaling Technology, USA), βTrCP (1:1000; Cat. No. F0415, Selleck Chemicals, USA), β-actin (1:20000; Cat. No. 66009, Proteintech, China), IκBα (1:1000; Cat. No. 10268-1-AP, Proteintech, China), HDAC1 (1:1000, Cat. No. ab109411, Abcam, UK), HDAC2 (1:1000, Cat. No. ab32117, Abcam, UK), acetyl-Histone3 (1:2000, Cat. No. ab52946, Abcam, UK), Ubiquitin (1:1000, Cat. No. 3936, Cell Signaling Technology, USA), γH2AX (1:500; Cat. No. 05-636, EMD Millipore, USA), and Cleaved caspase-3 (1:1000, Cat. No. 9661, Cell Signaling Technology, USA). The primary antibodies were incubated at 4 °C overnight. The secondary antibodies were incubated at room temperature for one hour including goat anti-mouse lgG (H + L) (1:5000, Cat. No. 31430, Thermo Fisher Scientific, USA) and goat anti-rabbit lgG (H + L) (1:5000, Cat. No. 31460, Thermo Fisher Scientific, USA). The relative protein intensities were quantified by ImageJ software.

For immunoprecipitation, the whole-cell lysate was mixed with IκBα antibody (1 µg/1 mg protein lysate, Cat. No. 10268-1-AP, Proteintech, China) at 4 °C overnight. Then, the protein A/G magnetic beads (Cat. No. HY-K0202, MedChemExpress, USA) were added to the lysate-antibody mixture at 4 °C for 4 h. After incubation, the magnetic beads were washed 4 times and collected using the magnetic separation device. Then they were denatured at 95 °C for 5 min. The remaining steps were the same as those in the Western blotting experiment.

### RNA isolation and real-time quantitative reverse transcriptase PCR

The RNA in animal tissues and cells was isolated by FastPure Cell/Tissue Total RNA Isolation Kit (Cat. No. RC112-01, Vazyme, China) and the concentration of RNA was quantified by NanoDrop ND-2000 spectrophotometer (Nadro Drop Technologies, Wilmington, DE, USA). Complementary DNA (cDNA) synthesis was performed using the HiScript III RT SuperMix for qPCR (+gDNA wiper) (Cat. No. R323-01, Vazyme, China). The real-time PCR was performed with the indicated primers, cDNA and Maxima SYBR Green (Cat. No. B21203, Selleck Chemicals, USA) on Light Cycler^®^ 480II (Roche Applied Science, Indianapolis, IN, USA). The Ct values (threshold cycle number) were acquired by the Light Cycler^®^ 480 Software release 1.5.0 SP4 software and analyzed using the 2^-△△Ct^ method. △△Ct = experimental group △Ct - control group △Ct, △Ct = (average Ct of the target gene of the control sample - average Ct of the control sample GAPDH). Primer sequences used in the study are listed in Supplemental **Supplementary Table 1**.

### Flow cytometry

All mice were humanely euthanized at the experimental endpoint, and breast tumors were harvested. Tumor single-cell suspensions were prepared using the Tumor Tissue Enzymatic Digestion Kit (Cat. No. NV002A-10, NuVita BIO, China) following the manufacturer’s instructions. The cell suspension was thoroughly washed with staining buffer (PBS + 2% Fetal Bovine Serum) and adjusted to 1 × 10^6^ cells/mL. Cells were then incubated with Fixable Viability Dye eFluor™ 780 (Cat. No. 65-0865, Thermo Fisher Scientific, USA) for 30 min at 4 °C in the dark. After washing with staining buffer, cells were resuspended in 100 μL diluted antibody working solution and incubated at 4 °C for 30 min in the dark to label surface markers. The surface antibodies used were as follows: FITC anti-mouse CD45 (Cat. No. 103108, BioLegend, USA), Brilliant Violet™ 786 anti-mouse CD11b (Cat. No. 417-0112-80, Thermo Fisher Scientific, USA), PE/Cyanine7 anti-mouse F4/80 (Cat. No. 123113, BioLegend, USA), APC anti-mouse CD86 (Cat. No. 159215, BioLegend, USA), and eFluor™ 506 anti-mouse MHC Class II (I-A/I-E) (Cat. No. 69-5321-82, Thermo Fisher Scientific, USA). For intracellular CD206 staining, cells were fixed and permeabilized using Cyto-Fast™ Fix/Perm Buffer Set (Cat. No. 426803, Biogend, USA) according to the manufacturer’s instructions. Briefly, cells were incubated with Cyto-Fast™ Fix/Perm Solution at room temperature for 20 min and rinsed with Cyto-Fast™ Perm Wash Solution. Cells were then incubated in 100 μL diluted PE anti-mouse CD206 (MMR) antibody working solution (Cat. No. 12-2061-82, Thermo Fisher Scientific, USA) at room temperature in the dark for 40 min. After thorough washing, cells were resuspended in staining buffer for detection. Data were acquired on a CytoFLEX S flow cytometer (Beckman Coulter, USA) and analyzed via FlowJo software. All statistical graphs were generated using GraphPad Prism software (Boston, Massachusetts USA).

### Isolation of bone marrow-derived macrophages

Mice were euthanized humanely, and their femurs and tibias were harvested after surface disinfection with 75% ethanol. Bone marrow was flushed out using a 1 mL syringe filled with pre-cooled PBS. The flushed bone marrow cells were filtered through a 70 μm nylon cell strainer (Cat. No. YA0961, Solarbio, China) to prepare single-cell suspensions. Bone marrow-derived monocytes were then cultured in RPMI 1640 medium (Cat. No. 12100046, MACGENE, China) supplemented with 10% Fetal Bovine Serum (Cat. No. 10270106, Gibco, USA) and 1% Penicillin-Streptomycin (Cat. No. V900929, Sigma-Aldrich, USA), followed by stimulation with 20 ng/mL macrophage colony-stimulating factor (M-CSF, Cat. No. C0042, Selleck Chemicals, USA) to generate pure macrophages. Cell treatments were initiated on Day 6.

### RNA-sequencing and bioinformatic analysis

Total RNA was extracted from DMBA-induced breast tumor tissues of the control and IR groups, with three parallel replicates per group. Quality control, library construction, and RNA sequencing were all performed at Aksomics Biotechnology Co., Ltd., Shanghai, China. The RNA-sequencing experiment and data analysis were consistent with our previous study (10). Differentially expressed genes were determined through statistical analysis (fold change > 1.5 and *p* < 0.05). Enrichment analysis was conducted using the R package clusterProfiler (version 3.14.3).

### Cell culture

MCF-7 (ATCC^®^ HTB-22™, RRID: CVCL_0031, ATCC, USA) and RAW264.7 cell lines (ATCC^®^ TIB-71™, RRID: CVCL_0493, ATCC, USA) were maintained in DMEM (Cat. No. CM15019, MACGENE, China) medium with 10% Fetal Bovine Serum (Cat. No. 10270106, Gibco, USA) and 1% Penicillin-Streptomycin (Cat. No. V900929, Sigma-Aldrich, USA). 4T1-luc cells (ATCC^®^ CRL-2539™, RRID: CVCL_0125, ATCC, USA) were maintained in 1640 medium (Cat. No. 12100046, MACGENE, China) with 10% Fetal Bovine Serum (Cat. No. 10270106, Gibco, USA) and 1% Penicillin-Streptomycin (Cat. No. V900929, Sigma-Aldrich, USA). The MCF-10A cell line (Cat. No. SCSP-575, Cell Bank of the Chinese Academy of Sciences, CSTR:19375.09.3101HUMSCSP575) was purchased from Stem Cell Bank (Chinese Academy of Sciences), maintained in DMEM/F12 medium (Cat. No. CM10092, MACGENE, China) supplemented with growth factors including 5% horse serum (Cat. No. 26050088, Gibco, USA), 100 ng/ml Cholera toxin (Cat. No. C8052, Sigma-Aldrich, USA), 20 ng/ml epidermal growth factor (Cat. No. E5036, Sigma-Aldrich, USA), 0.5 μg/ml hydrocortisone (Cat. No. 614157, Sigma-Aldrich, USA), 10 μg/ml human insulin (Cat. No. I9278, Sigma-Aldrich, USA), and 1% penicillin-streptomycin (Cat. No. V900929, Sigma-Aldrich, USA). Cell lines were passaged every 2 to 3 days. The number of passages of all cells before use is within five generations. All cells were validated to be mycoplasma-free and maintained at 37 °C in 5% CO_2_.

### Co-culture

Firstly, the tumor cells were inoculated into the culture dish. After adhering, we pretreated the cells with 500 µM VPA (Cat. No. P4543, Sigma-Aldrich, USA) for 24 h. Then, the cells were exposed to 8 Gy of X-rays (X-RAD225 OptiMAX, Pxi, USA) and incubated for 6 h before extracting proteins or RNA for detection. Meanwhile, the conditioned medium (CM) of the treated cells was centrifuged and the supernatant was collected. Subsequently, the CM was added to RAW264.7 cells at a 1:1 ratio with fresh medium. After 24 h, the RNA of macrophages was extracted. RAW264.7 cells were collected using PBS and lysed by repeatedly freezing and thawing in liquid nitrogen. Subsequently, the lysis solution was mixed with fresh culture medium at a ratio of 2:3, and then added to treat tumor cells. CCK-8 was used to assess cell survival.

0.2 mM QNZ (Cat. No. S4902, Selleck Chemicals, USA) was used to pre-treat the tumor cells for 24 h before VPA and IR treatment. 20 mM of the βTrCP inhibitor, GS143 (Cat. No. HY-110261, MedChemExpress, USA), was used to pre-treat tumor cells for 8 h before VPA and IR treatment.

### siRNAs and transfections

By transfecting 150 pmol of siRNA or an equal amount of non-targeting control siRNA (Genepharma, China) with Opti-MEM and Lipofectamine 2000 (Cat. No. 12566014, Thermo Fisher Scientific, USA) into MCF-7 cells, the knockdown of βTrCP, HDAC1 and HDAC2 was achieved. Sequences of siRNAs used in this study: si*HDAC1*-1: CAGCGACUGUUUGAGAACC, si*HDAC1*-2: CUAAUGAGCUUCCAUACAA, si*HDAC2*-1: GCGGAUAGCUUGUGAUGAA, si*HDAC2*-2: GCAAAGAAAGCUAGAAUUG; si*βTrCP*: AAGUGGAAUUUGUGGAACAUC. After 48 h, the transfection mixture was removed and the cells were treated with VPA and/or IR. The effective knockdown effect was confirmed by Western blotting.

### Cycloheximide chase experiment and MG132 experiment

After adherent cells were treated with 500 µM VPA for 24 hours, they were irradiated with 8 Gy X-rays and incubated for 6 h. Subsequently, 50 μg/mL cycloheximide (CHX, Cat. No. C1988, Sigma-Aldrich, USA) was added, and cellular proteins were collected at 0, 2, 4, and 8 h for Western blot analysis.

Alternatively, after VPA and IR treatment, cells were incubated with 20 μM MG132 (Cat. No. S2619, Selleck Chemicals, USA) for 12 h before cellular proteins were harvested for Western blot analysis.

### CCK8 assay

The cells were seeded at a density of 1000–2000 per well in a 96-well plate. After adhering to the plate, cells were treated with the macrophage lysate and then incubated for 48 h. Next, the fresh culture medium was replaced, and CCK8 solution (Cat. No. K1018, APExBIO, USA) was added and incubated at 37 °C for 4 h. The absorbance of each group’s solutions was measured at 450 nm using an enzyme immunoassay analyzer (Tecan Infinite^®^ M200 PRO, Switzerland).

### Colony formation assay

Tumor cells were cultured in 6-well plates and treated with IR (8 Gy), VPA or macropgahe lysate. After 14 days of incubation, colonies were fixed with 4% paraformaldehyde and stained using crystal violet (Cat. No. C8470, Solarbio, China). The plates were then photographed, and the surviving fraction was calculated.

### Statistical analysis

The data are expressed as mean ± standard deviation (SD). Statistical analyses were performed using GraphPad Prism 8.0 (GraphPad Software, Boston, Massachusetts USA). For comparing independent samples, the unpaired two-tailed Student’s *t* test was used; for comparing multiple groups, one-way analysis of variance (ANOVA) was employed. Results with a *P* value less than 0.05 were considered statistically significant. The *P* values were designated as: *, *P* < 0.05; **, *P* < 0.01, indicating a statistically significant difference.

## Results

### VPA enhances breast tumor radiosensitivity via macrophage polarization

**Figure 1:**
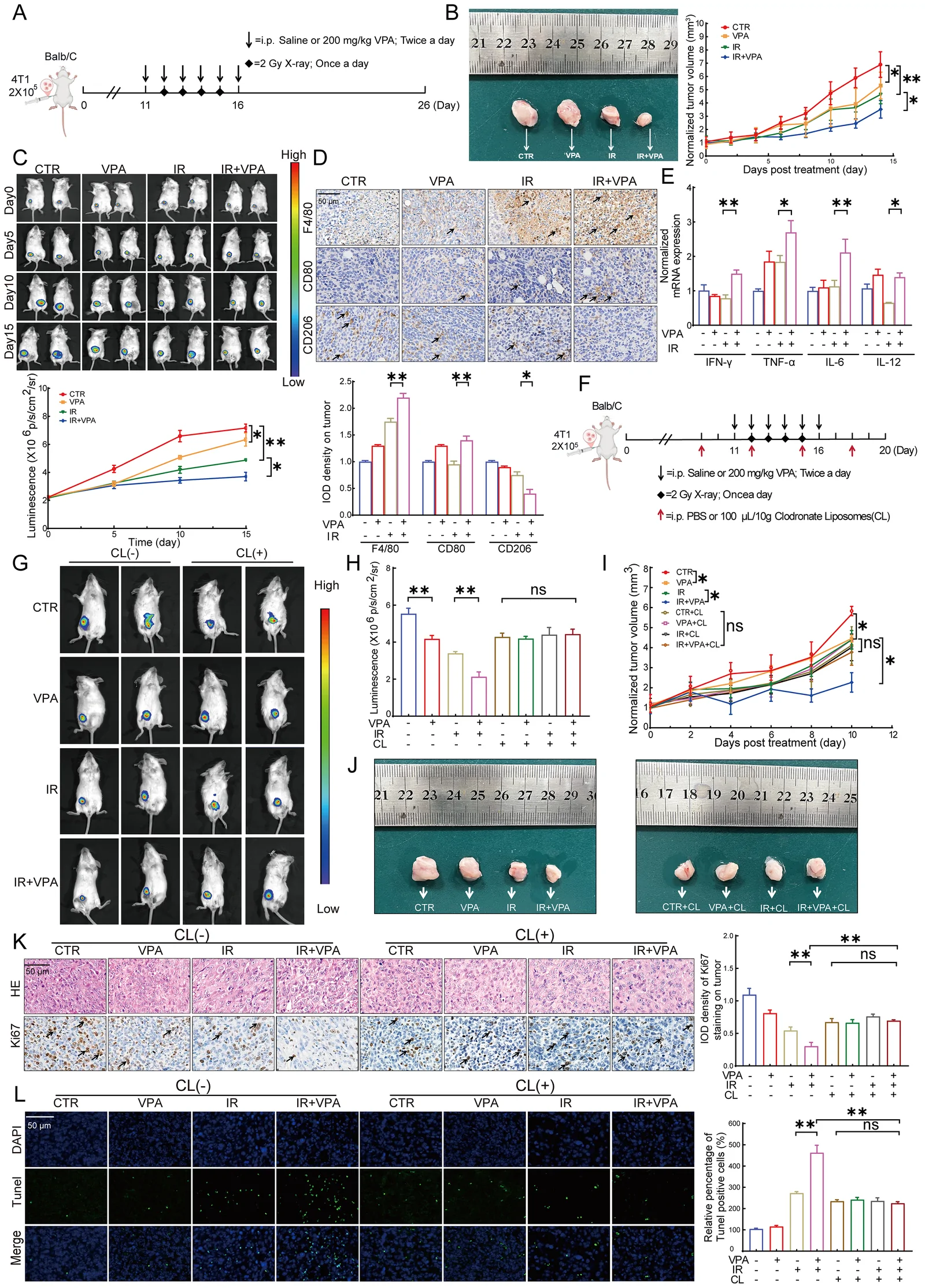
Valproic acid (VPA) enhances breast tumor radiosensitivity via macrophage polarization. **(A)** The schematic diagram of the 4T1-Luc-induced mouse breast transplant tumor model. 200 mg/kg of VPA was injected intraperitoneally (*i.p.*) twice a day for 6 consecutive days before and after the daily 2 Gy ionizing radiation (IR) for 4 consecutive days. **(B)** Morphological observation and normalized tumor volume of mouse breast tumors in each group (n=5 per group). **(C)** Bioluminescence imaging. **(D)** The representative IHC staining for macrophages and surface markers (F4/80, CD80, and CD206) in mouse tumor tissue sections from each group (scale bar, 50 µm). **(E)** The mRNA expression levels of inflammatory factors in mouse tumor tissues from each group were detected by RT-qPCR. **(F)** Schematic diagram of the establishment of the 4T1-Luc-induced mouse breast transplant tumor macrophage depletion model. Macrophages were depleted by clodronate liposomes (CL) (100 µL/10g, *i.p.*) every three days before and after treatments. The control group was administered with phosphate-buffered saline (PBS) (*i.p.*) (n=5 per group). **(G, H)** Bioluminescence images were captured, and the corresponding signals from mouse breast tumor sites in the macrophage-depletion model were quantified. **(I, J)** Morphological observation and normalized tumor volume in each group. **(K)** The representative image showed HE and proliferation marker Ki67 staining in mouse tumor tissue sections from each group (scale bar, 50 µm). **(L)** Tunel staining representing cell apoptosis levels of the mouse breast tumor tissues in each group (scale bar, 50 µm). Each data point in the graphs was from three independent experiments (mean ± SD). The *P* value was calculated using the Student’s t-test for the comparison of two independent samples and one-way ANOVA for the comparison of multiple groups (*P < 0.05, **P < 0.01).

To investigate the impact of VPA on breast tumor radiosensitivity *in vivo*, we established an orthotopic mouse breast xenograft model using 4T1-Luc cells (**Figure 1A**). Through morphological observation and continuous monitoring by bioluminescence imaging, treatment with 200 mg/kg VPA and 8 Gy IR resulted in a significant reduction in breast tumor volume and growth rate (**Figures 1B, C**). Histological analysis revealed significant disruption of the tumor architecture and increased vacuolation (**Supplementary Figure S1A**). The tumor tissue in the untreated group exhibited a dense and disorganized architecture, with tight cell junctions and almost no vacuolation. However, after combined treatment, the tumor cells showed a markedly loosened and disordered arrangement. Meanwhile, an increase in cytoplasmic vacuolation and expansion of vacuole structures in the combined treatment group was observed, accompanied by nuclear pyknosis and fragmentation, indicating severe damage and apoptosis of tumor cells. IHC demonstrated decreased proliferation markers, BrdU and Ki67, and increased apoptotic cells (TUNEL-positive cells) in the combined treatment tumor tissues (**Supplementary Figures S1A, B**). These results demonstrate that in the mouse breast transplant tumor model, VPA+IR treatment has a strong anti-tumor activity and enhances the radiosensitivity of breast tumors.

Next, to understand whether the mechanism underlying VPA-induced tumor radiosensitivity in breast tumors is related to activated immune responses. The macrophage marker F4/80 was detected by IHC. There was an increased infiltration of macrophages in tumor tissues after treatment (**Figure 1D**). Then, the polarization state of macrophages was detected. IHC showed that there was a notable shift in macrophage polarization towards the M1 phenotype, with elevated surface marker CD80 expression and reduced surface marker CD206 of the M2 phenotype (**Figure 1D**). Meanwhile, RT-qPCR showed upregulation of pro-inflammatory factors including IFN-γ, TNF-α, IL-6 and IL-12 secreted by M1 macrophages (**Figure 1E**). Next, we used flow cytometry to detect the infiltration of macrophages in the mouse tumor tissues. M1 macrophages were defined as CD45^+^ F4/80^+^ CD11b^+^ CD86^+^ MHC-II^+^ cells, whereas M2 macrophages were identified as CD45^+^ F4/80^+^ CD11b^+^ CD206^+^ cells. Results showed that the proportion of M1 macrophages gradually increased along with the IR+VPA treatment. Although the percentage of M2 macrophages showed a decreasing trend only in the VPA treatment group, the ratio of M1/M2 macrophages gradually increased under both IR or VPA treatments, and this increase was particularly significant in the combined treatment group (**Supplementary Figure S1C**). These results indicate that under VPA combined treatment, increased infiltration of M1 macrophages can be observed in the breast tumor tissues. These suggest that VPA’s radiosensitizing effect in breast tumors is associated with active immune responses induced by M1 macrophages.

*In vitro* studies of 4T1 and MCF-7 tumor cell lines further supported these findings. CM derived from 4T1 cells was applied to RAW264.7 macrophages for 24 h to simulate the impact of ITME on macrophage polarization (**Supplementary Figure S2A**). RT-qPCR was used to detect the mRNA expression levels of macrophage surface markers (CD86 and CD163), inflammatory (IL-12 and TNF-α) and anti-inflammatory factors (IL-10 and TGF-β). Results showed that CM from the VPA+IR treatment increased the ratio of M1 to M2 macrophage surface markers (CD86/CD163) (**Supplementary Figure S2B**) and enhanced the secretion of pro-inflammatory factor IL-12 (**Supplementary Figure S2B**), while reducing the secretion of anti-inflammatory factor TGF-β (**Supplementary Figure S2B**). Although TNF-α and IL-10 decreased slightly after treatments, the changes were not statistically significant. A similar result was also observed with MCF-7 cells (**Supplementary Figure S2D**). These results indicate that *in vitro* studies, the combined treatment can also promote the polarization of macrophages towards M1 phenotype. Subsequently, to observe the killing effect of M1 macrophages against tumor cells, the lysates of polarized macrophages were used to incubate tumor cells for 48 h. The CCK8 results showed that lysates from M1 macrophages significantly reduced tumor cell viability (**Supplementary Figures S2C, E**). Next, we isolated mouse BMDMs to establish a co-culture system to assess clonogenic survival for further verification of the observed phenomenon (**Supplementary Figure S2F**). We collected CM from 4T1 tumor cells, then cultured with primary BMDMs to obtain polarized macrophage-lysate. Tumor cells were subsequently incubated with macrophage lysate, followed by colony formation staining and quantitative counting. RT-qPCR was used to measure M1/M2 marker mRNA expression in BMDMs stimulated with tumor cell CM. Consistent with the RAW264.7 results, VPA/IR pre-treatment of tumor cells induced robust M1 polarization and elevated pro-inflammatory cytokine secretion in primary BMDMs (**Supplementary Figure S2G**). The clonogenic radiosensitivity assays showed that compared with the control group, M1 polarized BMDMs lysate markedly reduced the clonogenic capacity of 4T1 cells, consistent with that of RAW264.7 cells (**Supplementary Figures 2H–K**). Collectively, these *in vivo* and *in vitro* results indicate that macrophages may play a crucial role in VPA’s radiosensitizing effect on breast tumor.

To confirm the validity of these findings, CL were used to deplete macrophages in 4T1 breast transplant tumor mice (**Supplementary Figure S3A**). First, changes in the monocyte proportion in the bone marrow were detected by flow cytometry to determine the injection pattern of depletion agents. The results showed that on the second day, the proportion of monocytes significantly decreased. But on the third day, there was a notable increase (**Supplementary Figure S3B**). Based on this observation, CL were administered every 3rd day to establish an *in vivo* macrophage-depleted mouse mammary transplant tumor model (**Figure 1F**). Depletion of macrophages was confirmed by flow cytometry and IHC experiments (**Supplementary Figures S3C, D**). In this model, morphological observation and bioluminescence imaging revealed that, without CL, the tumor volume continued to decline after combined treatment. However, macrophage depletion abrogated the reduction in breast tumor volume. Among the CL groups, there was no longer any difference in tumor volume, and all were larger than the combined treatment group without CL (**Figures 1G–J**). The tumor weight showed the same trend (**Supplementary Figure S3E**). Meanwhile, macrophage depletion reversed the structural damage, decreased proliferation cell ratio and increased apoptotic cell ratio in the tumor tissues induced by VPA+IR treatment (**Figures 1K, L**). These phenomena suggest that after macrophage depletion, the effect of VPA to kill tumor tissue is suppressed, indicating that macrophages are essential mediators of VPA-induced radiosensitization.

Next, we established a macrophage-depletion-spontaneous-rescue animal model to provide complementary functional evidence (**Supplementary Figure S3F**). Based on the macrophage-depleted mouse mammary transplant tumor model, we added CL rescue groups except for CL (–) and CL (+) groups. In the CL rescue cohort, CL injection was delivered throughout the early therapeutic stage; subsequently, CL intervention was discontinued to allow endogenous macrophages to infiltrate and differentiate in tumor tissues. Results showed that in the CL (-) group, combined treatment exerted markedly stronger tumor growth suppression than IR alone. In contrast, sustained macrophage depletion in the CL (+) group completely abolished VPA’s radiosensitizing capacity, consistent with our earlier *in vivo* observations. Importantly, after the macrophage recovered in the CL rescue group, the anti-tumor effect of IR+VPA combination therapy was partially restored (**Supplementary Figures S3G, H**). These data suggest that loss of macrophages eliminates VPA’s radiosensitization, and restoration of endogenous macrophages rescues this phenotype. Collectively, it confirms that macrophages act as indispensable mediators of VPA-induced tumor radiosensitization.

### VPA’s radiosensitization is dependent on NF-κB-macrophages-mediated immune responses

**Figure 2:**
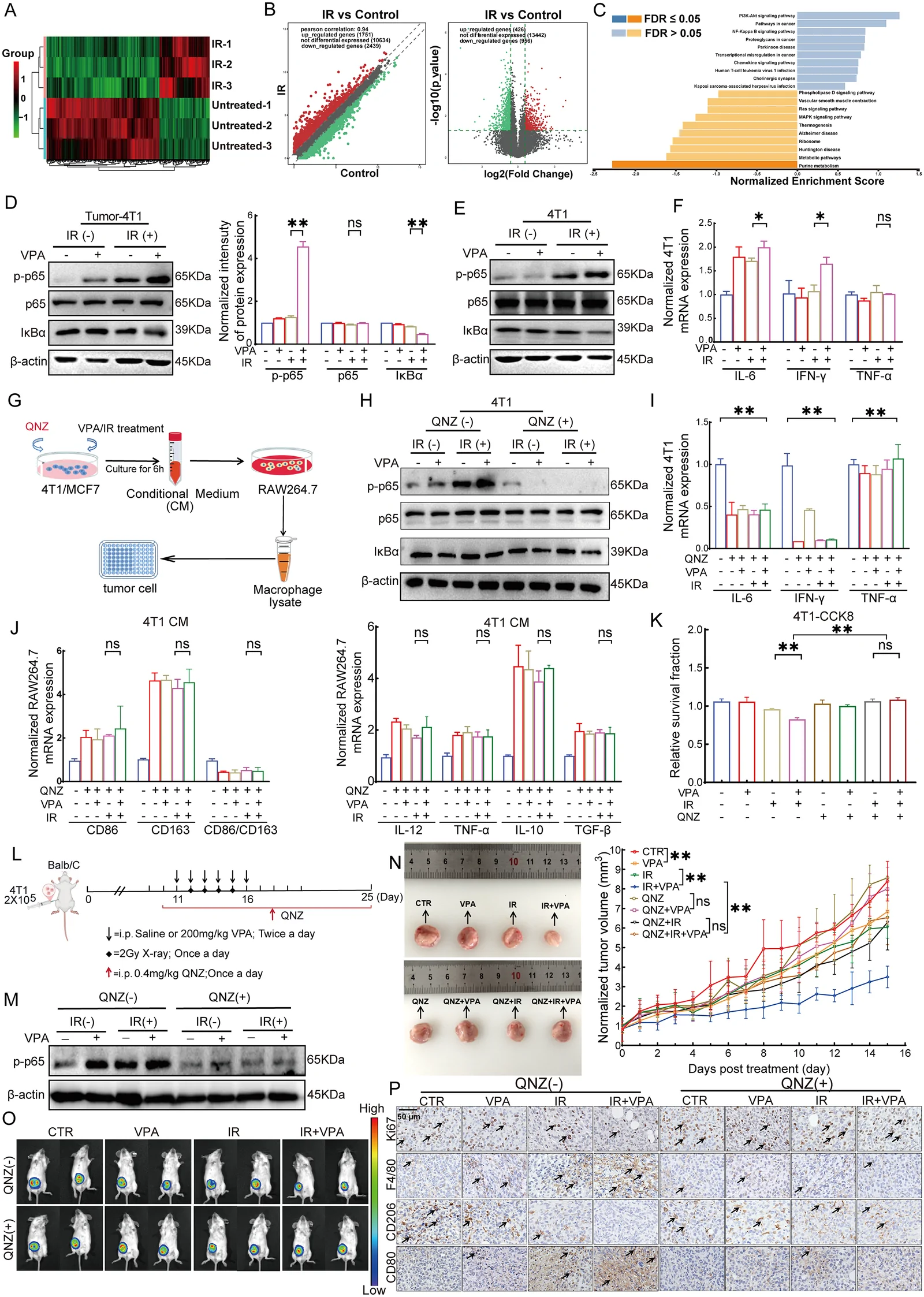
VPA’s radiosensitization is dependent on nuclear factor kappa B (NF-κB)-macrophage-mediated immune responses. **(A)** The heatmap showed a large number of changed genes between the IR and untreated groups (n=3 per group). **(B)** The scatter and volcano graph indicated significantly changed genes. **(C)** Enrichment analysis of significantly changed genes. **(D)** The main protein levels of NF-κB pathway in 4T1-Luc-induced mouse tumor tissues. **(E)** The main protein levels of NF-κB pathway in 4T1 cells. **(F)** The mRNA expression levels of key inflammatory factors were detected by RT-qPCR in 4T1 cells. **(G)** The schematic diagram showed the use of 0.2 µM QNZ (NF-κB activity inhibitor) *in vitro*. **(H, I)** The activity of NF-κB pathway detected by Western blotting and RT-qPCR in 4T1 cells after pre-treating with QNZ for 24 h. **(J)** RT-qPCR detected the polarization state of macrophages incubated with conditioned medium (CM) from 4T1 cells pre-treated with QNZ. **(K)** CCK8 was used to assess the growth status of 4T1 cells after treatment with macrophage lysate for 48 h. **(L)** Schematic diagram of the establishment of the 4T1-Luc-induced mouse breast transplant tumor QNZ inhibitor model (n=5 per group). **(M)** Western blotting to confirm the active state of phosphorylated p65 (p-p65) in the mouse tumor tissue. **(N)** Morphological observation and normalized tumor volume in each group of mice. **(O)** Bioluminescence images of tumor site were captured in the QNZ inhibitor model. **(P)** The representative image showed IHC staining for Ki67, F4/80, CD206, and CD80 in mouse tumor tissue sections from each group (scale bar, 50 µm). Each data point in the graphs was from three independent experiments (mean ± SD). The *P* value was calculated using the Student’s t-test for the comparison of two independent samples and one-way ANOVA for the comparison of multiple groups (*P < 0.05, **P < 0.01).

To explore the mechanism of VPA-induced radiosensitization on tumor tissues through regulation of the macrophage activity, we did RNA-seq of tumor tissues. We found that there were 1751 up-regulated genes and 2439 down-regulated genes in the IR-alone group compared to the untreated group, including 426 significantly up-regulated genes and 956 significantly down-regulated genes (**Figures 2A, B**). According to the KEGG database analysis of up-regulated genes, we found that multiple inflammation-related pathways were activated after IR treatment compared with the untreated group; all are closely related to the NF-κB inflammatory signaling pathway (**Figure 2C**). To verify the RNA-seq results, we used Western blotting to detect the main protein levels of the NF-κB inflammatory pathway, including p65, phosphorylated NF-κB p65 (p-p65), and IκBα, in breast tumor tissues induced by 4T1. Consistent with RNA-seq results, compared with the untreated group, the p-p65 protein level was increased in the IR-alone group, confirming that the NF-κB-mediated pathway was activated. Moreover, the additional treatment with VPA further elevated p-p65 expression, while the IκBα expression was inhibited with combined VPA+IR treatment. Meanwhile, the total protein expression level of p65 remained consistent across all treatment groups (**Figure 2D**). We conducted a further verification in DMBA-induced rat breast tumor tissues and observed the same trend (**Supplementary Figure S4A**). These *in vivo* results suggest that the activation of NF-κB-mediated inflammatory pathway may play a crucial role in VPA-induced radiosensitization in breast tumors. Subsequently, we performed further *in vitro* validation using MCF-7 and 4T1 cells. Consistent with the *in vivo* findings, combined VPA and IR treatment markedly activated the NF-κB pathway in both cell lines (**Figure 2E**; **Supplementary Figure S4B**) and elevated the secretion of pro-inflammatory cytokines IL-6, IFN-γ and TNF-α (**Figure 2F**; **Supplementary S4C**). Meanwhile, no significant difference was observed for TNF-α in 4T1 cells. Collectively, these data support the proposition that VPA enhances the radiosensitivity of breast tumors through the NF-κB-mediated inflammatory responses.

We next performed *in vitro* experiments to explore whether NF-κB signaling regulated macrophage polarization (**Figure 2G**). We pretreated 4T1 cells with 0.2 μM NF-κB inhibitor QNZ for 24 h to suppress NF-κB activity. Western blot results revealed that QNZ treatment markedly reversed the activation of the NF-κB pathway induced by combined treatment (**Figure 2H**). Correspondingly, pro-inflammatory factors were also significantly reduced compared with the untreated group after QNZ treatment (**Figure 2I**). After NF-κB activity was suppressed, the CM from 4T1 cells was used to incubate macrophages, we found that QNZ treatment abolished the M1 polarization induced by VPA+IR (**Figure 2J**). RT-qPCR results showed that upon QNZ, the ratio of macrophage surface markers CD86/CD163 and the expression levels of inflammatory factors, including IL-12, TNF-α, IL-10 and TGF-β, showed no statistically significant changes, even in the combined treatment group. Meanwhile, we observed that the macrophage lysate without QNZ treatment had a killing effect on 4T1 cells after combined treatment. But this phenomenon was reversed when QNZ was applied (**Figure 2K**). The above result was also observed in the MCF-7 cell line (**Supplementary Figures S4E–H**). As shown above, the suppression of NF-κB activity impaired the subsequent inflammatory response, inhibited the M1 macrophages polarization and reduced its ability to kill tumor cells. These *in vitro* results verify that the radiosensitizing effect of VPA is contingent upon NF-κB-mediated macrophage polarization.

To further verify these findings *in vivo*, QNZ was administered to maintain the NF-κB pathway in a suppressive state before and after treatment in the 4T1 breast transplant tumor mouse model. The dosage and administration method of QNZ (0.4 mg/kg once a day for 15 days) were determined through a preliminary experiment (**Supplementary Figure S4I**) and the schematic diagram was outlined in **Figure 2L**. Western blotting was used to assess NF-κB pathway activity. Our results showed that after QNZ application, p-p65 activity in the tumor tissue significantly decreased (**Figure 2M**). Meanwhile, the tumor volume continued to decrease after the combined treatment without QNZ. But this phenomenon was reversed after QNZ was applied (**Figure 2N**). After QNZ treatment, the combined treatment failed to suppress the tumor growth, and tumor volumes were notably larger than those in the VPA+IR group without QNZ. Bioluminescence imaging and tumor weight measurement also showed the same trend (**Figure 2O**; **Supplementary S4J, K**). The IHC results indicated that inhibition of the NF-κB pathway reversed the decrease in the proliferation marker Ki67 in the combined treatment group (**Figure 2P**; **Supplementary S4L**). These results indicate that inhibition of the NF-κB pathway attenuates the anti-tumor effect of VPA. In addition, in the group without QNZ, the combined treatment still promoted macrophage infiltration and reprogrammed them into the M1 phenotype; however, after QNZ intervention, this effect was significantly reversed. IHC staining showed no statistically significant differences in macrophage infiltration and polarization between the QNZ+IR+VPA and QNZ+IR groups. Compared with the IR+VPA group without QNZ administration, F4/80 and CD80 expression were markedly decreased, while CD206 remained highly expressed (**Figure 2P**; **Supplementary S4L**). These results indicate that the activity of the NF-κB pathway in tumor tissues regulates the macrophage polarization and the anti-tumor immune response. Consistent with earlier *in vitro* findings, these *in vivo* results futher demonstrate that VPA’s radiosensitizing effect in tumor tissues is dependent on NF-κB-mediated reprogramming of activated macrophages.

### βTrCP-mediated inflammatory response pathway is critical for VPA-induced radiosensitivity

**Figure 3:**
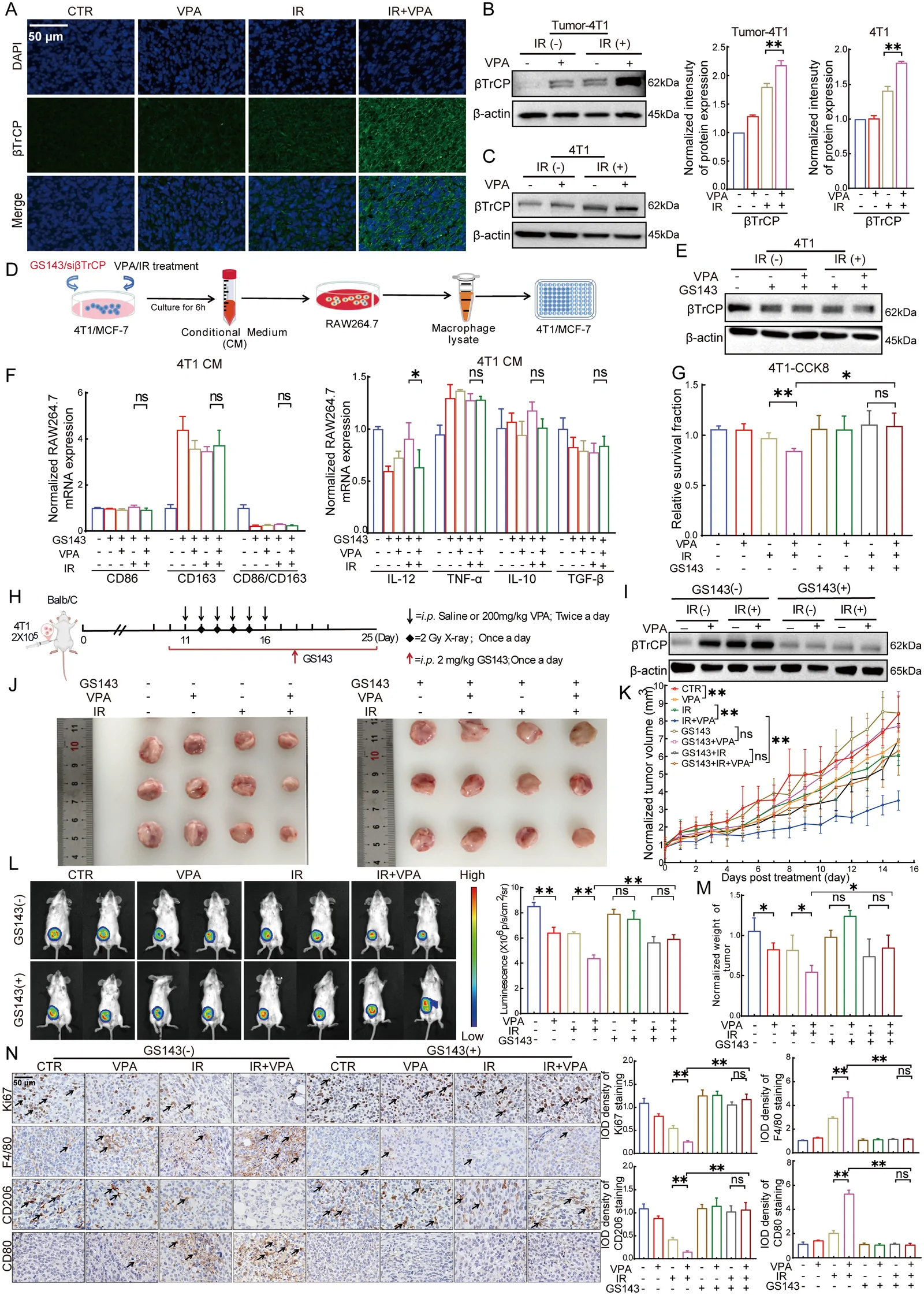
β-transducin repeat-containing protein (βTrCP)-mediated inflammatory response pathway is critical for VPA-induced radiosensitivity. **(A, B)** The expression level of βTrCP in 4T1-Luc-induced mouse tumor tissues was detected by western blotting and IF. **(C)** The level of βTrCP in 4T1 cells was detected by western blotting. **(D)** The schematic diagram showed the use of 20 µM GS143 (βTrCP inhibitor) or SiβTrCP to inhibit βTrCP expression *in vitro*. **(E)** Western blotting detected the protein level of βTrCP in 4T1 cells after pre-treatment with GS143 for 8 h. **(F)** RT-qPCR detected the polarization state of macrophages incubated with CM from 4T1 cells pre-treated with GS143. **(G)** CCK8 was used to assess the growth status of 4T1 cells after treatment with macrophage lysate for 48 h. **(H)** Schematic diagram of the establishment of the 4T1-Luc-induced mouse breast transplant tumor inhibitor model (n=5 per group). **(I)** Western blotting to confirm the inhibition state of βTrCP in the mouse tumor tissue. **(J, K)** Morphological observation and normalized tumor volume in each group of mice. **(L)** Bioluminescence images were captured, and the corresponding signals from mouse breast tumor sites in the GS143 inhibitor model were quantified. **(M)** Statistical analysis of the normalized tumor weight of each group of mice. **(N)** The representative image showed IHC staining for Ki67, F4/80, CD206, and CD80 in mouse tumor tissue sections from each group (scale bar, 50 µm). Each data point in the graphs was from three independent experiments (mean ± SD). The *P* value was calculated using the Student’s t-test for the comparison of two independent samples and one-way ANOVA for the comparison of multiple groups (*P < 0.05, **P < 0.01).

Given βTrCP’s role as an E3 ubiquitin ligase that targets IκBα for degradation and subsequently activates the NF-κB inflammatory signaling pathway, its involvement in VPA-mediated effects was next examined. To verify our hypothesis, we performed Western blotting and immunofluorescent staining of mouse tumor tissues to assess βTrCP expression. IR alone could elevate βTrCP levels, while the combination treatment further significantly upregulated βTrCP relative to IR alone treatment (**Figures 3A, B**). Similar results were observed in rat tumor tissues (**Supplementary Figure S5A**). *In vitro*, the combined treatment also increased βTrCP protein expression in the 4T1 and MCF-7 cells (**Figure 3C**; **Supplementary S5B**). Collectively, these data reveal that combined treatment significantly upregulates βTrCP expression both *in vivo* and *in vitro*. Considering the function of βTrCP in triggering IκBα degradation and NF-κB cascade activation, our results indicate that βTrCP may act as a critical mediator of VPA-induced radiosensitization.

To further investigate whether βTrCP was involved in VPA-induced radiosensitization through macrophage polarization, tumor cells were treated with the specific βTrCP inhibitor, GS143, *in vitro* to simulate changes in ITIME (**Figure 3D**). The preliminary experiment determined the optimal GS143 dosage to be 20 µM (**Supplementary Figure S5C**). When 4T1 cells were treated with GS143 for 8 h, we observed that GS143 reversed the increased expression of βTrCP caused by the combined VPA+IR treatment (**Figure 3E**). Subsequently, the CM of 4T1 cells was applied to incubate macrophages, and we found that the polarization to M1 phenotype macrophages after VPA+IR treatments was suppressed by GS143 (**Figure 3F**). The ratio of CD86/CD163 was no longer elevated and the pro-inflammatory factors secreted by macrophages were no longer increased after combined treatment. In addition, the killing effect of the macrophage lysate on 4T1 cells was also reversed (**Figure 3G**). The above results were also observed in the MCF-7 cell line when treated with SiβTrCP (**Supplementary Figures S5D–F**). Consistent with our observations of the NF-κB pathway, suppression of βTrCP expression disrupted M1 macrophage polarization and impaired their tumor-killing capacity. These *in vitro* findings confirm that VPA relies on βTrCP activity to regulate macrophage polarization and exert radiosensitizing effect.

To validate the above results *in vivo*, we also used GS143 to inhibit βTrCP before and after treatment in the 4T1 breast tumor transplant mouse model. The treatment dose and time interval for GS143 were determined in a preliminary experiment (**Supplementary Figure S5G**) and the final schematic diagram (2 mg/kg once a day for 15 days) were shown in **Figure 3H**. After GS143 treatment, VPA+IR no longer increased βTrCP protein expression in mouse tumor tissues, indicating that βTrCP activity was successfully inhibited (**Figure 3I**). We observed that tumor volume decreased significantly after combined treatment. After GS143 treatment, the tumor volume was no longer significantly reduced and was greater than that in the combined treatment group without GS143 intervention (**Figures 3J, K**). Bioluminescence imaging and tumor weight measurement also showed the same trend (**Figures 3L, M**). The IHC results indicated that inhibition of the βTrCP pathway could reverse the reduction in the proliferation marker Ki67 induced by combined treatment. Accordingly, inhibition of βTrCP activity abrogated the anti-tumor effects of VPA. Meanwhile, we performed IHC staining to evaluate macrophage infiltration. In the absence of GS143, the combination treatment further elevated M1 macrophage infiltration relative to the IR-only group. However, GS143 administration markedly reversed this phenotype. No statistically significant differences in macrophage infiltration and polarization were detected after GS143 administration, and the infiltration of M1 macrophages was significantly lower than that in the GS143-free combined treatment group (**Figure 3N**). Collectively, these *in vivo* and *in vitro* results indicate that the radiosensitizing effect of VPA on tumor tissues depend on the activated βTrCP-mediated pathway and the subsequent reprogramming of TAMs.

### VPA modulates protein stability and ubiquitination of IκBα via βTrCP

**Figure 4:**
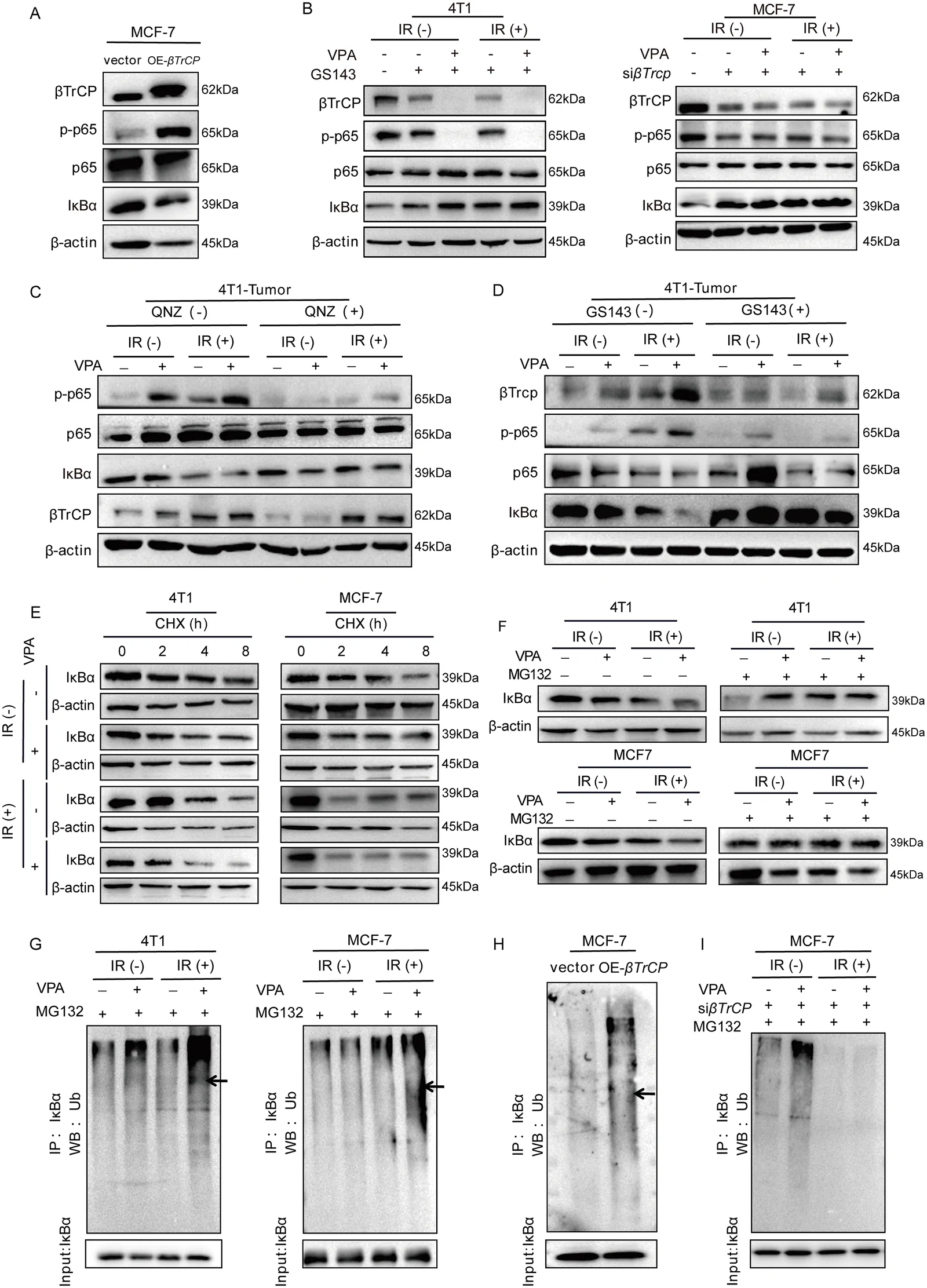
VPA modulates protein stability and ubiquitination of NF-κB inhibitor alpha (IκBα) via βTrCP. **(A)** The expression levels of the main proteins of the NF-κB pathway were detected using western blotting when βTrCP was overexpressed in MCF-7 cells. **(B)** The expression levels of the main proteins of the NF-κB pathway were detected using western blotting after βTrCP was inhibited in 4T1 and MCF-7 cells. **(C)** The expression levels of βTrCP in the 4T1-Luc-induced mouse breast transplant tumor QNZ inhibitor model. **(D)** The expression levels of the main proteins of the NF-κB pathway in the 4T1-Luc-induced mouse breast transplant tumor GS143 inhibitor model. **(E)** Using 0.5 mM VPA and 8 Gy IR to treat 4T1 and MCF-7 cells, and then the protein stability of IκBα was detected by western blotting at 0, 2, 4, and 8 h after treatment with cycloheximide (CHX) (50 µg/ml). **(F)** After treating 4T1 and MCF-7 cells with 0.5 mM VPA and 8 Gy IR, the protein expression of IκBα was detected after 12 h of MG132 (proteasome inhibitor) treatment (20 µM). **(G)** The protein ubiquitination level of IκBα by immunoprecipitation in 4T1 and MCF-7 cells after treatment with 0.5 mM VPA and 8 Gy IR. **(H)** The protein ubiquitination level of IκBα by immunoprecipitation in MCF-7 cells after overexpression of βTrCP. **(I)** The protein ubiquitination level of IκBα by immunoprecipitation in MCF-7 cells after inhibiting βTrCP.

To investigate whether the VPA-mediated effects depend on the association between βTrCP and the NF-κB pathway, we first overexpressed βTrCP in MCF-7 cells and observed that elevated βTrCP levels were accompanied by robust activation of the NF-κB signaling pathway (**Figure 4A**; **Supplementary Figure S6A**). When we knocked out βTrCP in 4T1 and MCF-7 cell lines, we observed that NF-κB activity was subsequently reduced, even after combined treatments (**Figure 4B**; **Supplementary Figure S6B**). Next, we conducted further verification using the *in vivo* model established previously. In the 4T1 tumor tissues, QNZ treatment did not reduce βTrCP protein expression when the NF-κB pathway was inhibited (**Figure 4C**; **Supplementary Figure S6C**). However, when GS143 was administered, the decrease in βTrCP expression subsequently led to the inhibition of the NF-κB pathway (**Figure 4D**; **Supplementary Figure S6D**). These results indicate a potential association between βTrCP and NF-κB demonstrating that βTrCP acts upstream of the NF-κB pathway to mediate anti-tumor immune responses during VPA-induced radiosensitizing effect.

Next, we sought to elucidate the mechanism by which VPA modulates the interaction between βTrCP and NF-κB to regulate the radiosensitivity of breast tumors. Given that βTrCP functions as an E3 ubiquitin ligase to ubiquitinate IκBα and trigger its proteasomal degradation, we hypothesized that IκBα serves as a critical mediator linking βTrCP to NF-κB pathway activation under VPA treatment. Therefore, we employed the protein synthesis inhibitor CHX to treat the 4T1 and MCF-7 tumor cell lines that had been previously exposed to VPA and IR. Protein samples were collected at 0, 2, 4, and 8 h following CHX treatment to assess the stability of IκBα. The Western blot results demonstrated that the combined VPA+IR treatment significantly reduced the protein stability of IκBα (**Figure 4E**; **Supplementary Figure S6E**). Next, we explored whether IκBα protein instability was associated with ubiquitination-dependent degradation. After treating tumor cells with the 26S proteasome inhibitor MG132, we found that it reversed the reduction in IκBα protein levels induced by VPA+IR (**Figure 4F**; **Supplementary Figure S6F**), and the immunoprecipitation assays revealed that VPA+IR markedly increased the ubiquitination of IκBα (**Figure 4G**; **Supplementary Figure S6G**). These results suggest that combined VPA and IR treatment promotes IκBα degradation via the βTrCP-dependent ubiquitin-proteasome pathway.

To further investigate, we overexpressed βTrCP in MCF-7 cells and observed a significant increase in IκBα ubiquitination (**Figure 4H**; **Supplementary Figure S6H**). Conversely, silencing βTrCP expression with siRNA in MCF-7 cells abolished the increase in IκBα ubiquitination, even under combined VPA+IR treatment (**Figure 4I**; **Supplementary Figure S6I**). These results confirm that the ubiquitination and degradation of IκBα in response to VPA combined IR treatment are regulated by βTrCP, and it is a key factor in the activation of the NF-κB inflammatory signaling pathway.

### VPA regulates the βTrCP-NF-κB signaling pathway by inhibiting HDAC1/2 expression

**Figure 5:**
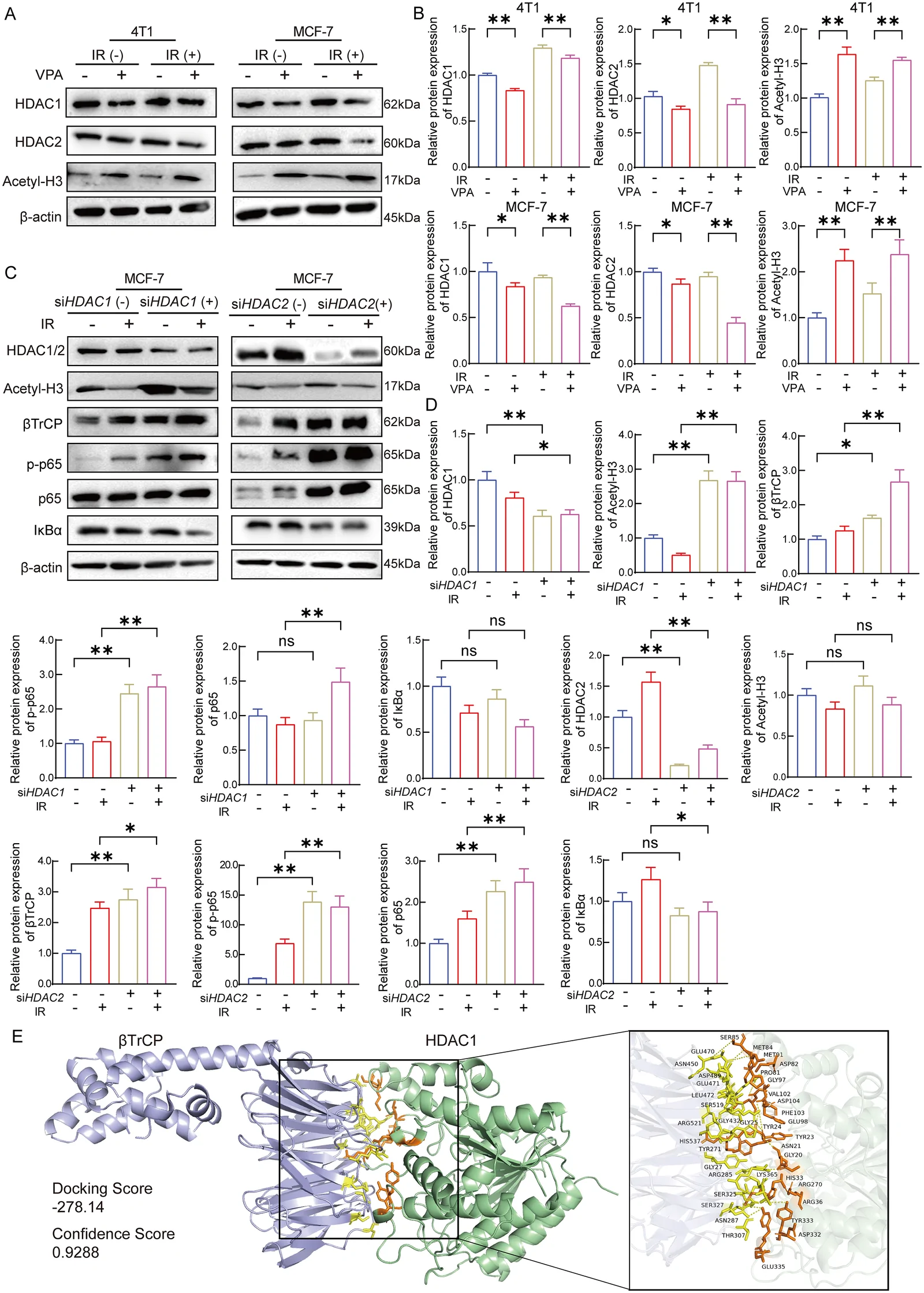
VPA regulates the βTrCP-NF-κB signaling pathway by inhibiting histone deacetylase 1/2 (HDAC1/2) expression. **(A, B)** Western blotting detected the protein levels of HDAC1/2 in 4T1 and MCF-7 cells after 0.5 mM VPA and 8 Gy IR. The statistical significances are included in **(B)** with * being *P* < 0.05, ** *P* < 0.01. **(C, D)** The protein expression levels of the βTrCP-NF-κB pathway were detected using western blotting after treating MCF-7 cells with SiHDAC1/2 and 8 Gy IR. The statistical significances are included in **(B)** with * being *P* < 0.05, ** *P* < 0.01. **(E)** The molecular docking of HDAC1 and βTrCP was used to present the protein binding.

Next, we investigated how VPA modulated the expression of βTrCP and the NF-κB inflammatory pathway. Since VPA is a class I and IIa HDACi, we first assessed HDAC1/2 protein levels in 4T1 and MCF-7 tumor cells. Western blot analysis revealed that after VPA and VPA+IR treatments, HDAC1/2 protein expression was markedly reduced compared with untreated controls and cells treated with IR alone. Conversely, histone H3 acetylation levels were increased in response to VPA (**Figures 5A, B**). This indicates that VPA treatment effectively suppresses HDAC1/2 expression in tumor cells.

To further investigate whether HDAC1/2 protein expression affects activation of the βTrCP pathway, we used si*HDAC1/2* in MCF-7 cells to knock out HDAC1/2, thereby simulating the effect of VPA treatment. This treatment increased βTrCP protein expression in both the HDAC1/2 knockdown group and the combined treatment group. Activation of the NF-κB signaling pathway was also observed (**Figures 5C, D**). These results verify that loss of HDAC1/2 is sufficient to drive βTrCP-NF-κB signaling activation, placing HDAC1/2 functionally upstream of βTrCP. In addition, we conducted molecular docking analysis between HDAC1 and βTrCP. The top-ranked docking model displays stable binding between HDAC1 and βTrCP with a favorable docking score of −278.41 and a confidence score of 0.9288. This structural simulation supports a physical association between HDAC1 and βTrCP at the molecular level (**Figure 5E**). Collectively, these findings suggest that VPA regulates the βTrCP-NF-κB immune pathway in breast tumor cells through the inhibition of HDAC1/2 expression.

### VPA alleviates RAE by reducing M1 macrophage infiltration in non-targeted normal tissues

**Figure 6:**
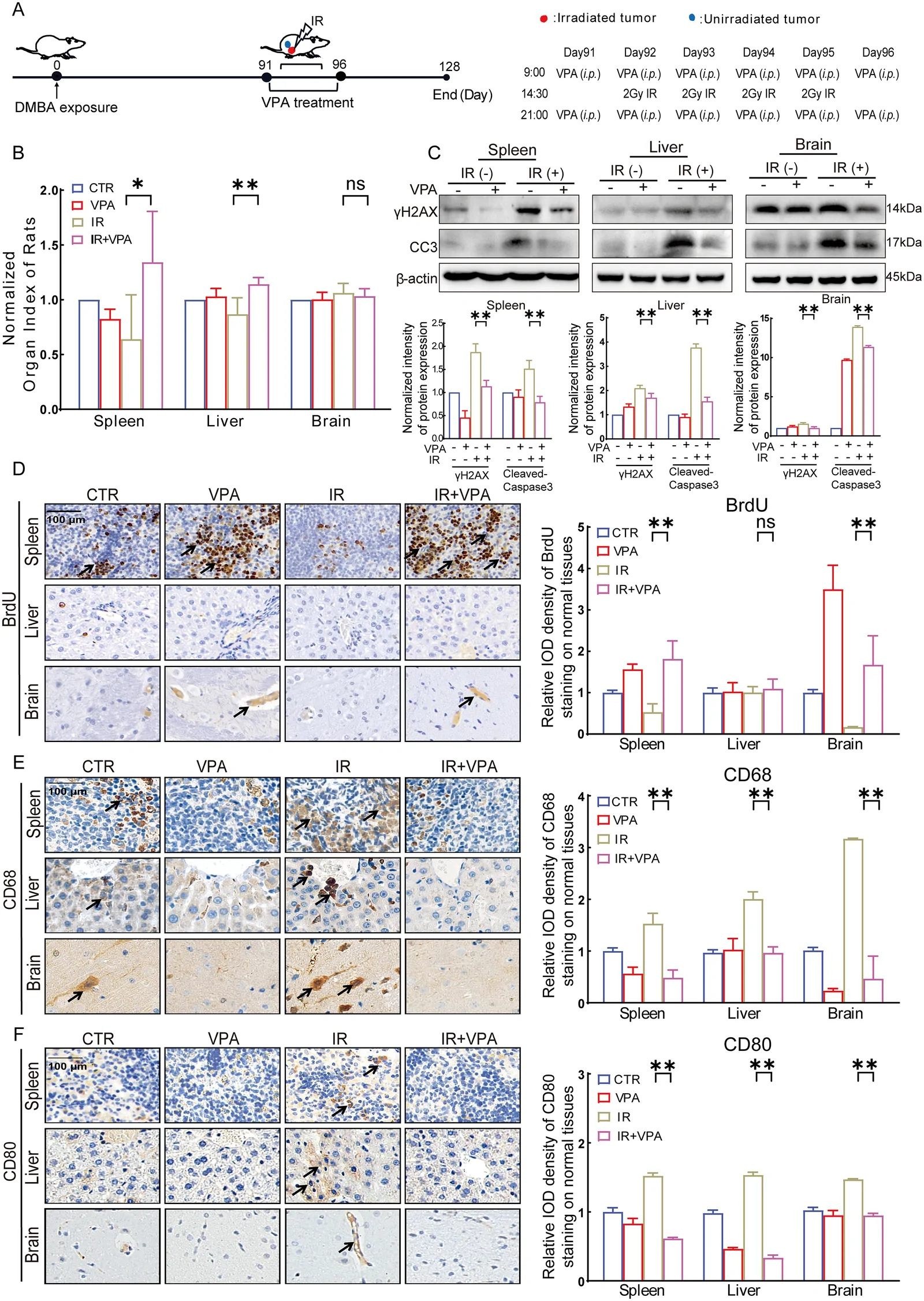
VPA alleviates abscopal effects (RAE) by reducing M1 macrophage infiltration in non-targeted normal tissues. **(A)** The schedule of VPA administration and 2 Gy × 4 days of IR treatment in the 7,12-dimethylbenz[a]anthracene (DMBA)-induced rat breast tumor RAE model. VPA was injected into rats twice a day for 6 days and performed before and after the daily 2 Gy IR. **(B)** The organ index (OI) of rat normal tissues was calculated by the formula ( $OI=\frac{Weight_{organ}}{Weight_{body}}$). **(C)** The protein expression of cell damage and apoptosis markers in the rat distant normal tissue was detected by western blotting. **(D–F)** The representative image showed IHC staining of BrdU, CD68 and CD80 in the rat distant normal tissue sections of each group (scale bar, 100 µm). The quantitative results as presented on the right side. Each data point in the graphs was from three independent experiments (mean ± SD). The *P* value was calculated using the Student’s t-test for the comparison of two independent samples (*P < 0.05, **P < 0.01).

Then, we established an RAE model using the DMBA-induced rat breast tumor animal model to further investigate the mechanisms by which VPA reduces IR damage in non-targeted normal tissues (**Figure 6A**). Initial observations revealed that VPA attenuated the reduction in organ indices (OI) of the spleen and liver caused by IR (**Figure 6B**). Although the OI of the brain tissue showed no significant changes, the Western blot results showed that VPA reversed the IR-induced elevation of DNA double-strand break (DSB) markers and apoptosis-specific proteins, such as γH2AX and cleaved caspase-3 (CC3) in the normal tissues (**Figure 6C**). IHC analysis also showed that VPA counteracted the IR-induced decrease in proliferation markers, BrdU (**Figure 6D**) and Ki67 in the spleen (**Supplementary Figure S6A**). Following VPA treatment, although the number of Ki67-positive cells in brain tissue did not change markedly, the count of BrdU-positive cells showed a clear upward trend. In liver tissue, BrdU-positive cell numbers increased only slightly though not statistically significance, while Ki67-positive cells exhibited a distinct upward trend. Collectively, these results indicate that VPA confers radioprotection in non-targeted normal tissues during RT.

To determine whether this observed radioprotective effect is associated with macrophage-mediated immune responses, we performed IHC staining for CD68 and F4/80 in normal tissues. VPA treatment significantly reduced the IR-induced increase in macrophage infiltration in the spleen, liver, and brain (**Figure 6E**; **Supplementary S6B**). Similarly, the myeloid-derived cell marker CD11b showed the same trend (**Supplementary Figure S6C**). We next investigated whether VPA exerts radioprotective effects on normal tissues by regulating macrophage polarization. IHC staining for M1 macrophage markers demonstrated that VPA markedly reversed the IR-induced increase in CD80-positive cells (**Figure 6F**). RT-qPCR analysis found that VPA enhanced the reduction in the CD163/CD86 ratio induced by IR in the spleen, liver and brain. VPA reversed the IR-induced upregulation of pro-inflammatory factors IFN-γ and IL-12. VPA also promoted the expression of anti-inflammatory factors TGF-β and IL-10 in the spleen, and increased TGF-β expression in the brain. No statistically significant changes were observed in the liver (**Supplementary Figure S6D**). Taken together, these results suggest that VPA may alleviate inflammatory responses and confer radioprotective effects in non-target normal tissues by reducing the infiltration of M1-polarized myeloid-derived macrophages.

### VPA alleviates RAE in non-targeted normal tissues via the βTrCP-NF-κB inflammatory pathway

**Figure 7:**
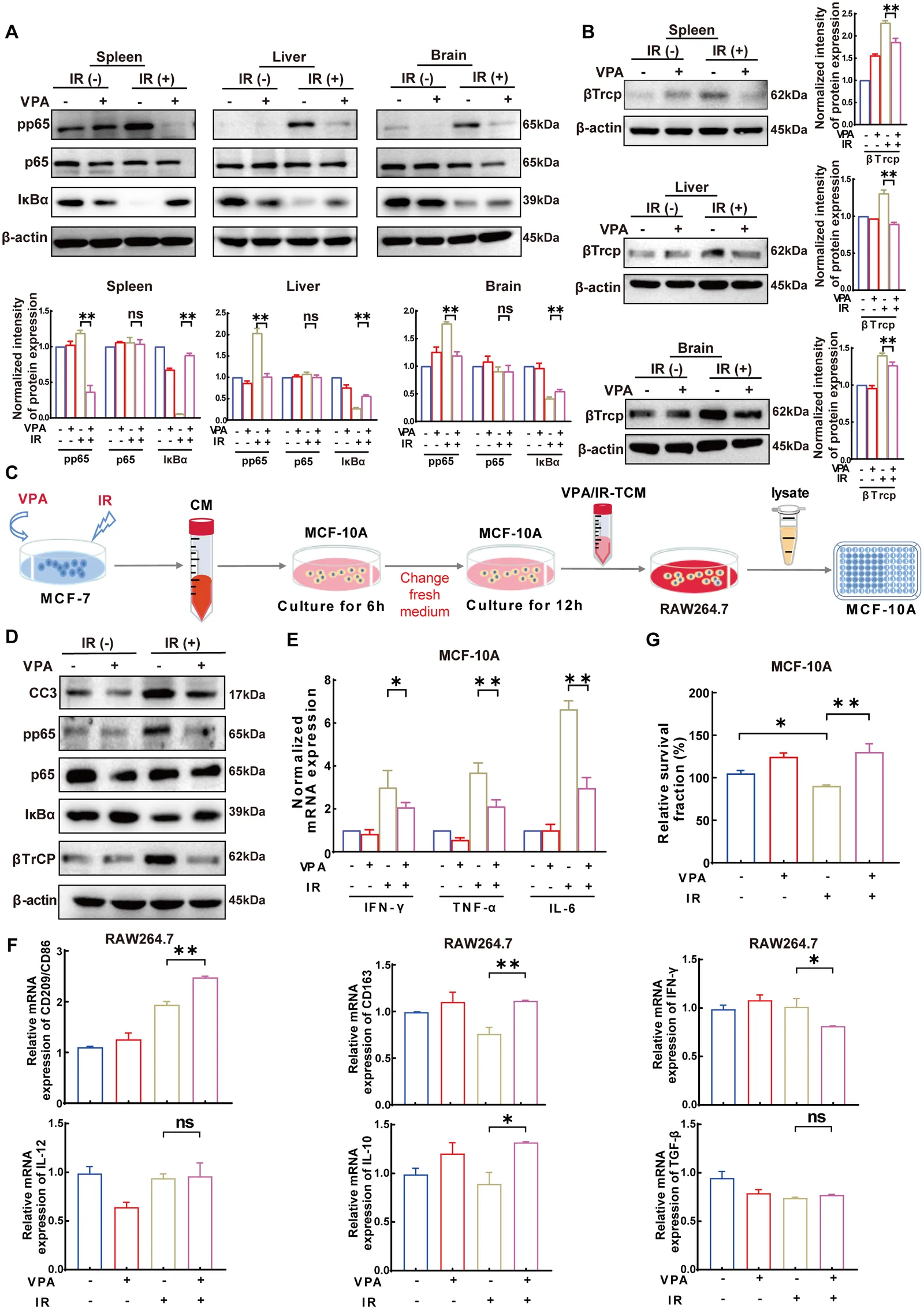
VPA alleviates RAE in non-targeted normal tissues via βTrCP-NF-κB inflammatory pathway. **(A)** The main protein levels of the NF-κB inflammatory pathway in the rat distant normal tissue were detected by western blotting. **(B)** The protein level of βTrCP in the rat distant normal tissue of each group was detected by western blotting. **(C)** The schematic diagram illustrates the simulation of RAE in non-targeted normal MCF-10A cells *in vitro*. **(D)** The protein expression levels of the βTrCP-NF-κB pathway were detected using western blotting in MCF-10A cells after incubation with CM from MCF-7 cells treated with 0.5 mM VPA and 8 Gy IR. **(E)** The mRNA expression levels of inflammatory factors were detected using RT-qPCR in MCF-10A cells after incubation with CM from MCF-7 cells treated with 0.5 mM VPA and 8 Gy IR. **(F)** The mRNA expression levels of the surface marker and inflammatory factors secreted by macrophages using RT-qPCR when incubated with VPA/IR-Treated Conditional Medium (VPA/IR-TCM) from MCF-10A cells. **(G)** CCK8 was used to assess the growth status of MCF-10A cells after treatment with macrophage lysate for 48 hours. Each data point in the graphs was from three independent experiments (mean ± SD). The *P* value was calculated using the Student’s t-test for the comparison of two independent samples (*P < 0.05, **P < 0.01).

To further elucidate the mechanism by which VPA mitigates RAE in non-targeted normal tissues, we investigated the role of βTrCP-NF-κB-macrophage-mediated inflammatory signaling pathway. Western blot results showed that VPA significantly decreased the p-p65 expression induced by IR and increased the IκBα protein level, while p65 levels showed no difference (**Figure 7A**). These results suggest that VPA attenuates NF-κB-mediated inflammatory responses, thereby alleviating RAE in normal tissues.

We further analyzed βTrCP protein levels in normal tissues. Western blot results showed that βTrCP expression was significantly upregulated by IR treatment, whereas this effect was markedly reversed by VPA treatment (**Figure 7B**). This regulatory effect was also observed in non-targeted tissues in our 4T1 mouse transplantation tumor RAE model (**Supplementary Figures S7A–E**). Findings support the notion that VPA’s protective action on normal tissues is associated with inhibition of the βTrCP-NF-κB-mediated immune response.

To validate these results, we used MCF-10A normal breast epithelial cells to model RAE in non-targeted normal cells. We first treated MCF-7 cells with 0.5 mM VPA before the 8 Gy IR. After 6 h, the CM from MCF-7 was transferred to MCF-10A cells (**Figure 7C**). Western blot was then performed to detect the protein levels of inflammatory response markers in MCF-10A cells. Results showed that VPA treatment led to reduced levels of apoptosis, p-p65, and βTrCP in MCF-10A cells compared to the IR-only group (**Figure 7D**), along with decreased mRNA expression of pro-inflammatory factors including IFN-γ, TNF-α and IL-6 (**Figure 7E**). These findings indicate that VPA attenuates the βTrCP-NF-κB-mediated inflammatory response in normal cells. To further dissect the pathway, we pre-treated MCF-7 cells with 20 μM GS143, followed by VPA and IR administration. The CM of the treated MCF-7 was then transferred to MCF-10A cells. Compared with the untreated group, GS143 significantly reduced βTrCP and p-p65 expression in normal cells. Moreover, even in the combined treatment group, the protein levels of these inflammatory markers did not show significant changes compared to the IR group (**Supplementary Figure S7F**). This indicates that the βTrCP-NF-κB-mediated inflammatory response played a crucial role in the radioprotective effect of VPA on the non-targeted normal tissues.

We next investigated whether VPA-mediated inhibition of the βTrCP-NF-κB pathway in MCF-10A cells could further affect macrophage polarization. Therefore, we simulated RAE *in vitro*. We incubated MCF-10A normal cells with the CM of treated MCF-7 cells for 6 h, then replaced the medium with fresh medium and continued culturing for another 12 h. The resulting VPA/IR-Treated Conditional Medium (VPA/IR-TCM) was then applied to RAW264.7 macrophages. RT-qPCR analysis showed that compared with the IR group, the ratio of M2/M1 macrophage surface markers CD209/CD86, CD163 and anti-inflammatory factor IL-10 significantly increased, while the pro-inflammatory factor IFN-γ decreased after VPA treatment (**Figure 7F**). These results indicate that VPA can inhibit the activation of the βTrCP-NF-κB-macrophage signaling pathway induced by IR treatment.

Finally, lysate from these polarized macrophages was applied to MCF-10A cells, resulting in a significant increase in cell proliferation compared to the IR group (**Figure 7G**). These results indicate that VPA protects normal cells by promoting macrophage polarization toward the M2 phenotype. Collectively, these findings highlight the critical role of the βTrCP-NF-κB-mediated inflammatory response in VPA’s radioprotective function in non-targeted normal tissues.

## Discussion

**Figure 8:**
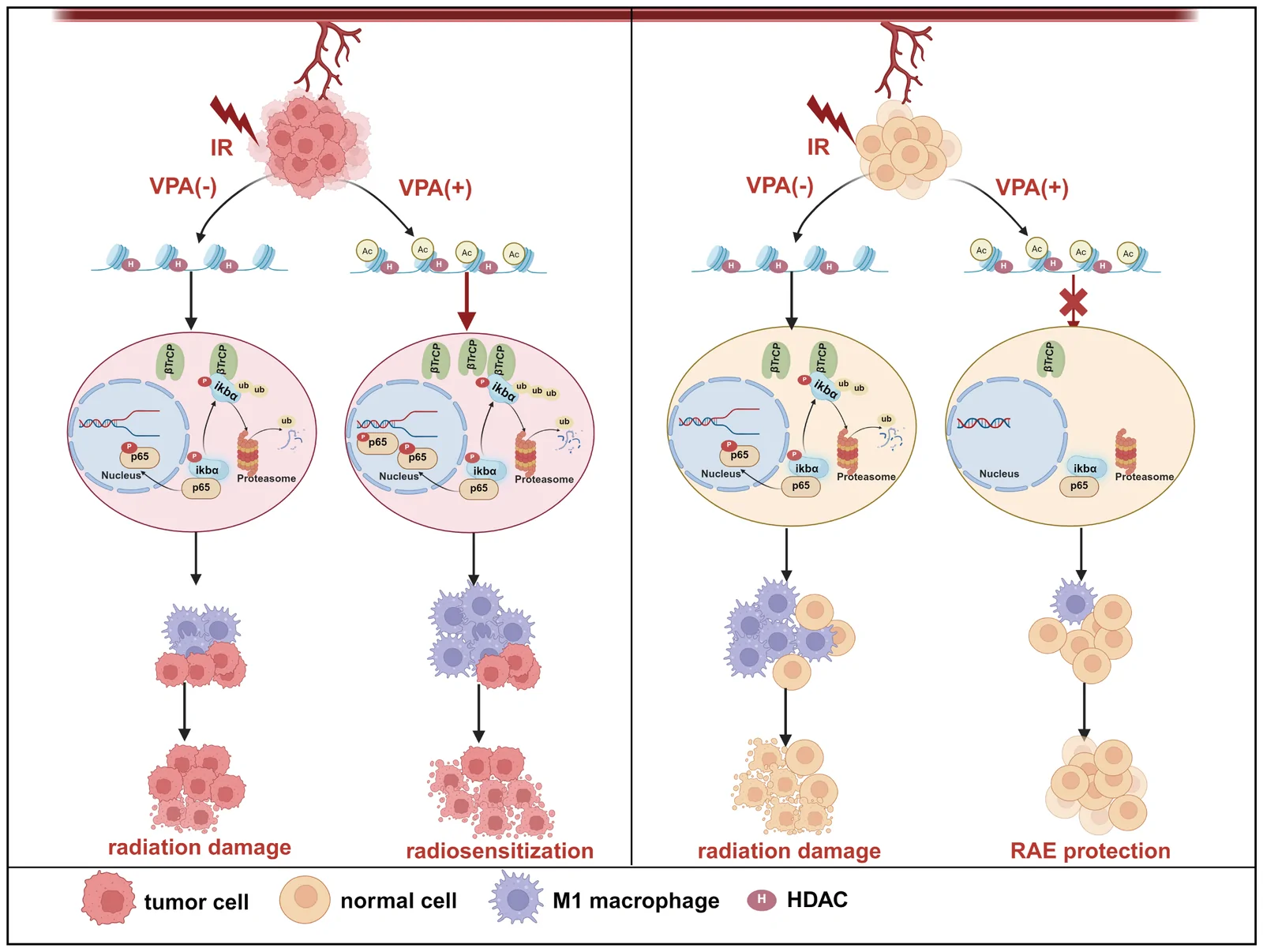
Schematic illustration showing the immune mechanism by which VPA exerts dual regulatory effects on tumor tissues and normal tissues under radiotherapy.

This study demonstrates that VPA exerts radiosensitization effect on breast tumors is closely linked to macrophage polarization towards the M1 phenotype, as validated by *in vivo* and *in vitro* experiments. VPA-mediated macrophage polarization is regulated via the βTrCP-NF-κB pathway. Specifically, VPA promotes βTrCP-mediated ubiquitination and degradation of the NF-κB inhibitory molecule IκBα, which facilitetes the activation and nuclear translocation of NF-κB p65. This signaling axis establishes a βTrCP-NF-κB-macrophages-mediated cascade response that underpins the anti-tumor immunity of VPA and enhances tumor radiosensitivity. Notably, the activation of the βTrCP-NF-κB pathway by VPA is achieved through suppression of HDAC1/2. In contrast to its effects on tumor tissues, VPA also alleviates RAE in non-targeted normal tissues by suppressing the activation of the βTrCP-NF-κB-macrophages signaling pathway, thereby conferring radioprotection (**Figure 8**). Collectively, these findings suggest that VPA exerts a bidirectional, tissue-dependent regulatory effects on tumor and normal tissues, supporting its potential as an adjuvant for clinal RT.

### VPA’s radiosensitization relies on macrophage-mediated anti-tumor immunity

The advent of immunotherapy has transformed oncology treatment by harnessing the body’s immune response to fight tumors (39), and activating immune function can turn “cold” tumors into “hot” tumors (40), a critical factor for effective cancer therapy. TAMs constitute a significant proportion of tumor-infiltrating immune cells, with M1 TAMs exerting tumor-suppressing effects and M2 TAMs promoting tumor progression (41). M2 TAMs foster immunosuppression and support tumor cell survival by inhibiting other immune cells, such as T cells and NK cells, thereby facilitating immune evasion (42–44). Conversely, M1 TAMs are effective in eliminating cancer cells and promoting T cells proliferation and IFN-γ release (45). Reprogramming M2 TAMs into M1 phenotype is a novel and promising strategy for enhancing anti-tumor immunity (46). In this study, VPA is identified as an efficacious adjuvant to RT, capable of reprogramming TAMs toward the M1 phenotype and increasing M1 macrophage infiltration in tumor tissues, particularly in the highly invasive 4T1 mouse xenograft model, which closely resembles triple-negative breast cancer (TNBC). The radiosensitizing effect of VPA is fundamentally linked to the activation of a macrophage-mediated anti-tumor immune response and to the effective conversion of “cold” tumors into “hot” tumors. These findings are consistent with previous work using the DMBA-induced rat breast tumor model, which also highlighted the anti-tumor effects of VPA via M1 macrophage activity (9, 47). Importantly, the 200mg/kg dose of VPA employed in our study aligns with therapeutic dosing for epilepsy and is included on the WHO Essential Medicines List, providing a strong rationale for its safe use as an RT adjuvant.

### The βTrCP-NF-κB-macrophage inflammatory axis serves as a novel mechanism underlying VPA-induced radiosensitization

Understanding the regulatory mechanism governing macrophage polarization is essential to elucidate the radiosensitizing effects of VPA. M1 macrophages are usually activated by pro-inflammatory stimuli such as IFN-γ, lipopolysaccharides (LPS), or GM-CSF (48–50), and this activation involves five distinct signaling pathways: PI3K/AKT, TLRs/NF-κB, JAK-STAT, Notch, and JNK (51, 52). Other mechanisms, such as cell signaling transduction, post-transcriptional regulation, epigenetic changes, and cellular metabolism, can also significantly influence the phenotype and function of immune cells (41). Our study found that the combination of VPA and IR treatment activated the βTrCP-NF-κB pathway to reprogram M1 polarization and modulated the TIME, thereby enhancing tumor radiosensitivity.

Mutiple lines of evidence support this regulatory mechanism. First, NF-κB is a central regulator of inflammation and is sensitive to DNA damage and oxidative stress induced by IR (53, 54). Recent mechanistic investigations of breast cancer therapy have further underscored the critical function of NF-κB signaling in mediating therapeutic resistance and tumor progression. One report illustrated PRMT1/PARP1-driven NF-κB activation impacts TNBC metastasis and chemoresistance (55). Activation of the NF-κB pathway leads to a pro-inflammatory state and promotes M1 polarization (56). Under hypoxic conditions, NF-κB can further reprogram macrophages to an inflammatory phenotype, countering the immunosuppressive TIME (57, 58). The interplay between NF-κB and HDAC has attracted attention, with HDACi entinostat shown to exert an anti-ovarian cancer effect by activating the NF-κB signaling pathway and mediating TNF-α secretion (59). VPA has been previously verified to affect the phosphorylation level of NF-κB p65 (29). These studies collectively support the indispensable role of NF-κB signaling in VPA-mediated tumor radiosensitization.

Second, βTrCP, as an E3 ubiquitin ligase, targets phosphorylated IκBα for proteasomal degradation, releasing the p65 subunit of NF-κB (35). Beyond NF-κB, βTrCP also regulates the ubiquitination of cell cycle factors in response to DNA damage responses (60). Previous studies have shown that βTrCP helps to enhance the T cell-dependent anti-tumor response and reduces tumor immune escape (61), as well as modulate IL-1β, TNF and IL-6 expression in macrophages (62). Based on these findings, our study systematically demonstrated that VPA facilitates IκBα ubiquitination and degradation in a βTrCP-dependent manner, which initiates robust NF-κB signaling activation and subsequently promotes M1 macrophage polarization and anti-tumor immune responses.

Moreover, HDACis (such as VPA) can upregulate E2 ubiquitin-binding enzymes and E3 ubiquitin ligase subunits, Ube2D1 and SKP1A in the SCF family, to be involved in steroidogenesis (63). Our study supported the correlation between HDAC1/2 inhibition and βTrCP function. However, the regulatory targets between the two still need to be further explored, including chromatin-level validation. In addition, similar to the natural small-molecule isoliquiritigenin reported in recent breast cancer research (64), VPA, as a synthetic small-molecule epigenetic modulator, exerts anti-tumor effects by targeting intracellular transcriptional signaling cascades to reverse radiotherapy resistance in breast cancer. Collectively, our findings establish the βTrCP-NF-κB inflammatory axis as a novel and critical mechanism responsible for VPA-mediated breast tumor radiosensitization.

### VPA protects non-targeted normal tissues from radiation injury via suppressing βTrCP-NF-κB signaling

The RAE of non-targeted normal tissues is a significant clinical challenge, often limiting the effectiveness of RT (5). The underlying mechanism of RAE is not fully understood, but IR-induced activation of the NF-κB pathway has been implicated in various IR injuries, including radiation-induced liver damage and neurogenesis impairment from whole-brain irradiation in rats (65, 66). In our study, fractionated IR treatment induced obvious RAE phenotypes in multiple normal organs, which was accompanied by the activation of the βTrCP-NF-κB pathway, predominant M1 macrophage polarization, and subsequent inflammatory tissue injury. There is a pressing need for developing novel agents that simultaneously enhance tumor radiosensitivity and alleviate RAE in normal tissues.

Recent research has highlighted the anti-inflammatory potential of HDACis in multiple inflammatory and tissue injury models (67), including spinal cord injury (68), renal inflammatory lesions (67), and ischemia-reperfusion lung injury (69). Consistent with these research findings, our results demonstrated the unique dual regulatory effects of VPA in tumor and normal tissues. Specifically, VPA enhances breast tumor radiosensitivity by activating the βTrCP-NF-κB-mediated pro-inflammatory response within the TIME, whereas it mitigates RAE in non-targeted normal tissues by inhibiting excessive βTrCP-NF-κB signaling and promoting M2 macrophage polarization. These findings highlight the tissue-specific bidirectional regulatory function of VPA, distinguishing it from conventional radiosensitizers and providing a novel theoretical basis for its clinical application as a dual-functional RT adjuvant.

### Limitations and future perspectives

Although this study uncovers a new immunological mechanism by which VPA exerts dual radiosensitizing and radioprotective effects, several experimental limitations and unresolved questions remain to be further explored. First, bulk RNA-seq performed in this study was based on only three biological replicates. Insufficient replicates may lead to missed detection of weakly expressed genes and introduce analytical bias (70). Second, the precise mechanisms underlying VPA’s differential effects in tumors versus normal tissues warrant further investigation. Third, the relationship between βTrCP and T cell-mediated immunity remains to be clarified, as previous work suggests that VPA can sustain macrophage-CD8^+^ T cell-mediated anti-tumor immune responses (9), and other HDACi, 2-hexyl-4-pentenoic acid (HPTA), can activate and recruit CXCR3^+^CD4^+^ T cells to the tumor tissues through the NF-κB-mediated CXCL9/10 pathway, assisting CD8^+^ T cells to exert anti-tumor functions (71). Given βTrCP’s roles in VPA-mediated inflammatory responses, whether VPA-regulated inflammation is linked to CD8^+^ and CD4^+^ T cell responses is an important area for future research. Furthermore, cell-cell communication (CCC) plays a vital role in the stability of the internal environment of organisms and disease progression (72). The ligand-receptor mode and transport carrier of exosomes are a common form of CCC. For instance, CHI3L1 secreted by glioma cells interacts with the transmembrane receptor CD44, thereby promoting the M2 phenotype (73). In recent years, advances in single-cell RNA sequencing technology (scRNA-Seq) have made it possible to explore the ligand-receptor interactions between tumor or normal cells and immune cells (74), providing deeper mechanistic insights and potential therapeutic targets for VPA-assisted clinical RT.

## Conclusions

In conclusion, our research demonstrates that VPA can simultaneously enhance tumor radiosensitivity and protect normal tissues from IR injury by modulating the βTrCP-NF-κB-macrophage inflammatory response in a bidirectional manner. This novel mechanism offers a strong experimental foundation for the use of HDACis as adjuvants to RT.

## Data Availability

The RNA-sequencing data generated in this study are available upon reasonable request from the corresponding author. All the other data generated or analyzed during this study are included in this published article and its supplementary information files.
