# Supplementary material for: Macrophage polarization regulated by the βTrCP-NF-κB inflammatory signaling pathway is required for valproic acid’s dual role in breast tumor radiosensitivity and normal tissue radioprotection
Source: Front Immunol. 2026 Aug 10;17:1839063. doi: 10.3389/fimmu.2026.1839063 (PMC13500527; doi:10.3389/fimmu.2026.1839063)
Supplement: Supplementary file 1 [file Table1.docx]

Supplementary Material

# Supplementary Data

# Supplementary Figures and Tables

# 1.1 Supplementary Figures

**Supplementary Figure S1.** **VPA enhances the radiosensitivity of breast tumor tissues by polarizing macrophages *in vivo*.** **(A)** The representative image showed HE and IHC staining for BrdU and Ki67 in mouse tumor tissue sections from each group (scale bar, 50 µm). The quantitative results as presented on the right side. The arrow points to the cells with positive expression.**(B)** Tunel staining representing cell apoptosis levels of the mouse breast tumor tissues in each group (scale bar, 50 µm). **(C)** Flow cytometry was used to detect the infiltration of macrophages in mouse tumor tissues.

**Supplementary Figure S2.** **VPA enhances the radiosensitivity of breast tumor tissues by polarizing macrophages *in vitro*.** **(A)** A schematic diagram for the establishment of the *in vitro* model for collecting the conditioned medium (CM) of two tumor cell lines, 4T1 and MCF-7, detection of macrophage polarization and tumor cell growth viability. **(B)** The mRNA expression levels of the surface marker and inflammatory factors secreted by macrophages using RT-PCR when incubated with CM from 4T1 cells. **(C)** CCK8 was used to assess the growth status of 4T1 cells after treatment with macrophage lysate for 48 h. **(D)** The mRNA expression levels of the surface marker and the inflammatory factors secreted by macrophages were measured by RT-PCR after incubation with CM from MCF-7 cells. **(E)** CCK8 was used to assess the growth status of MCF-7 cells after treatment with macrophage lysate for 48 h. **(F)** A schematic diagram of the *in vitro* model. Polarized macrophages lysate was collected to incubate 4T1 tumor cells and detect clonogenic survival. **(G)** RT-qPCR analysis detected the bone marrow-derived macrophages (BMDMs) polarization after 24 h incubation with the CM of 4T1 cells treated with VPA and IR. **(H-I)** Colony formation assays and corresponding quantitative statistics assessing clonogenic survival of 4T1 cells treated with lysate from BMDMs. **(J-K)** Corresponding quantitative statistics of colony formation assay in Colony formation assays showing survival of 4T1 cells cultured with lylate from RAW264.7. Each data point in the graphs was from three independent experiments (mean ± SD). The *P* value was calculated using the Student's t-test for the comparison of two independent samples and one-way ANOVA for the comparison of multiple groups (**P* < 0.05, ***P* < 0.01).

**Supplementary Figure S3. Establishment of the 4T1-Luc-induced mouse breast transplant tumor macrophage depletion model.** **(A)** Schematic diagram of the pre-experiment on the injection pattern of clodronate liposomes (CL, 100 µL/10 g, *i.p.)* to tumor-bearing mice. Samples were collected on day 0, 2, and 3 after the injection. **(B)** Flow cytometry was used to detect changes in the proportion of bone marrow monocytes. **(C)** The proportion of monocytes in the bone marrow was measured by Flow cytometry in the 4T1-Luc-induced mouse breast transplant tumor macrophage-depletion model. **(D)** The representative image of IHC staining on F4/80 in the tumor tissue sections of the 4T1-Luc-induced mouse breast transplant tumor macrophage depletion model (scale bar, 50 µm). The quantitative results as presented on the right side. **(E)** Statistical analysis of the standardized mouse tumor weight in the 4T1-Luc-induced mouse breast transplant tumor macrophage depletion model. **(F)** Schematic diagram of the establishment of the macrophage depletion-spontaneous rescue animal model. **(G-H)** Morphological observation and normalized tumor volume in each group of the spontaneous macrophage rescue animal model. Each data point in the graphs was from three independent experiments (mean ± SD). The *P* value was calculated using the Student's t-test for the comparison of two independent samples and one-way ANOVA for the comparison of multiple groups (**P* < 0.05, ***P* < 0.01).

**Supplementary Figure S4. The radiosensitizing effect of VPA on tumor tissues depends on NF**-κB**-macrophage-mediated immune responses. (A)** The main proteins level of the NF-κB inflammatory pathway in DMBA-induced rat breast tumor tissues detected by western blotting (n=3 per group). **(B)** The main protein levels of the NF-κB inflammatory pathway in MCF-7 cells were detected by western blotting. **(C)** The mRNA expression levels of key inflammatory factors were detected by RT-PCR in MCF-7 cells. **(D)** The inhibitory effect of QNZ on p-p65 activity at low to high doses was detected by western blotting in 4T1 cells. Finally, the intermediate dosage of 0.2 µM was selected. **(E)** Western blotting detected the main protein levels of the NF-κB inflammatory pathway in MCF-7 cells after pre-treating with QNZ for 24 h. **(F)** The mRNA expression levels of key inflammatory factors were detected by RT-qPCR in MCF-7 cells after pre-treating with QNZ for 24 h. **(G)** RT-qPCR detected mRNA expression levels of the surface marker and inflammatory factors secreted by macrophages incubated with CM from MCF-7 cells pre-treated with QNZ. **(H)** CCK8 was used to assess the growth status of MCF-7 cells after treatment with macrophage lysate for 48 h. **(I)** 0.4 mg/kg and 1 mg/kg QNZ (*i.p.*) were administered to tumor-bearing mice, and samples were collected on day-1 and -2 after the injection. WB detected the inhibitory effect of QNZ on the p-p65 activity in mouse tumor tissues. Finally, the 0.4 mg/kg dosage was selected for daily continuous injection. **(J-K)** Statistical analysis of the standardized mouse tumor weight and bioluminescence intensity of each group in the mouse tumor QNZ inhibitor model. **(L)** The quantitative results of IHC staining on Ki67, F4/80, CD80 and CD206 in tumor tissues of each group. Each data point in the graphs was from three independent experiments (mean ± SD). The *P* value was calculated using the Student's t-test for the comparison of two independent samples and one-way ANOVA for the comparison of multiple groups (**P* < 0.05, ***P* < 0.01).

**Supplementary Figure S5. VPA enhanced radiosensitivity of breast tumors by regulating the βTrCP-mediated inflammatory response pathway. (A)** The main protein level of the βTrCP in DMBA-induced rat breast tumor tissues was detected by western blotting (n=3 per group). **(B)** The level of βTrCP in MCF-7 cells was detected by western blotting. **(C)** Western blotting detected the inhibitory effect of GS143 on the βTrCP at dosages of 10, 15 and 20 µM in 4T1 cells. The 20 μM dose had a significant inhibitory effect. **(D)** Western blotting detected the protein level of βTrCP in MCF-7 cells after SiβTrCP treatment. **(E)** RT-qPCR detected mRNA expression levels of the surface marker and inflammatory factors secreted by macrophages incubated with CM from MCF-7 cells treated with SiβTrCP. **(F)** CCK8 was used to assess the growth status of MCF-7 cells after treatment with macrophage lysate for 48 h. **(G)** 2 mg/kg, 10 mg/kg and 18 mg/kg GS143 (*i.p.*) were administered to tumor-bearing mice, and samples were collected on day-1 and -2 after the injection. WB detected the inhibitory effect of GS143 on the βTrCP in mouse tumor tissues. The 2 mg/kg dosage was selected for daily continuous injection. Each data point in the graphs was from three independent experiments (mean ± SD). The *P* value was calculated using the Student's t-test for the comparison of two independent samples and one-way ANOVA for the comparison of multiple groups (**P* < 0.05, ***P* < 0.01).

**Supplementary Figure S6.** Statistical analysis of the protein expression levels of each group in Figure 4. The *P* value was calculated using the Student's t-test for the comparison of two independent samples and one-way ANOVA for the comparison of multiple groups (**P* < 0.05, ***P* < 0.01).

**Supplementary Figure S7. VPA alleviated RAE by reducing infiltration of M1 macrophages into non-targeted normal tissues.** **(A-C)** The representative image showed IHC staining for Ki67, F4/80, and CD11b in rat distant normal tissue sections from each group (scale bar, 100µm). The quantitative results as presented on the right side. **(D)** RT-qPCR detected mRNA expression levels of the macrophage surface marker and secreted inflammatory factors in rat distant normal tissues. Each data point in the graphs was from three independent experiments (mean ± SD). The arrow points to the cells with positive expression. The *P* value was calculated using the Student's t-test for the comparison of two independent samples (**P* < 0.05, ***P* < 0.01).

**Supplementary Figure S8. VPA alleviated RAE through regulating the βTrCP-NF**-κB **inflammatory signaling pathway in non-targeted mouse normal tissues.** **(A)** The representative image showed HE staining of the mouse distant normal tissue sections of each group (scale bar, 100 µm). **(B-C)** The representative image showed IHC staining for BrdU and F4/80 in mouse distant normal tissue sections from each group (scale bar, 50µm). The quantitative results as presented on the right side. Each data point in the graphs was from three independent experiments (mean ± SD). The *P* value was calculated using the Student's t-test for the comparison of two independent samples (**P* < 0.05, ***P* < 0.01). The arrow points to the cells with positive expression. **(D)** The main proteins level of the NF-κB inflammatory pathway in the mouse distant normal tissue detected by western blotting. **(E)** The proteins level of βTrCP in the mouse distant normal tissue of each group was detected by western blotting. **(F)** The protein expression levels of the βTrCP**-**NF-κB pathway were detected using western blotting in MCF-10A cells after incubation with CM from MCF-7 cells pre-treated with 20 μM GS143 before 0.5 mM VPA and 8 Gy IR.

## **1.2** Supplementary Table

Table S1 The primer sequences used in this study

| Oligo Name(Rat) | Primer Sequence (5’-3’) |
| --- | --- |
| GAPDH | Forward: AGT GCC AGC CTC GTC TCA TA |
|  | Reverse: GAT GGT GAT GGG TTT CCC GT |
| CD86 | Forward: AAG CCC GTG TCC TTG ATC TG |
|  | Reverse: AGA CAT GTG TAA CCT GCA CCA T |
| IFN-γ | Forward: ACA ACC CAC AGA TCC AGC ACA AAG |
|  | Reverse: CAC CGA CTC CTT TTC CGC TTC C |
| IL-12 | Forward: CCT CAA GTT CTT CGT CCG CAT CC |
|  | Reverse: CAT TGG ACT TCG GCA GAG GTC TTC |
| CD163 | Forward:AGC ATG GCA CAG GTC ATT CA |
|  | Reverse: GGT CAC AAA ACT TCA ACC GGA |
| TGF-β | Forward:GAC CGC AAC AAC GCA ATC TAT GAC |
|  | Reverse: CTG GCA CTG CTT CCC GAA TGT C |
| IL-10 | Forward:CTG CTC TTA CTG GCT GGA GTG AAG |
|  | Reverse: TGG GTC TGG CTG ACT GGG AAG |
| Oligo Name(Mouse) | Primer Sequence (5’-3’) |
| GAPDH | Forward: GGT TGT CTC CTG CGA CTT CA |
|  | Reverse: GGT GGT CCA GGG TTT CTT ACT C |
| CD86 | Forward: ACG GAG TCA ATG AAG ATT TCC T |
|  | Reverse: GAT TCG GCT TCT TGT GAC ATA C |
| TNF-α | Forward: ATG TCT CAG CCT CTT CTC ATT C |
|  | Reverse: GCT CCT CCG CTT GGT GGT TTG |
| IFN-γ | Forward: CTT GAA AGA CAA TCA GGC CAT C |
|  | Reverse: CTT GGC AAT ACT CAT GAA TGC A |
| IL-12 | Forward: TGA GAA GTA TTC AGT GTC CTG C |
|  | Reverse: CTG TGA GTT CTT CAA AGG CTT C |
| CD163 | Forward: GTT TGT GGA GCC ATT CTA TTG G |
|  | Reverse: GGA AAC TGT AAG TCG CTG AAT C |
| TGF-β | Forward: CCA GAT CCT GTC CAA ACT AAG G |
|  | Reverse: CTC TTT AGC ATA GTA GTC CGC T |
| IL-10 | Forward: TTC TTT CAA ACA AAG GAC CAG C |
|  | Reverse: GCA ACC CAA GTA ACC CTT AAA G |
| Oligo Name(Human) | Primer Sequence (5’-3’) |
| GAPDH | Forward: GGAGCGAGATCCCTCCAAAAT |
|  | Reverse: GGCTGTTGTCATACTTCTCATGG |
| TNF-α | Forward: AGCCCTGGTATGAGCCCATCTATC |
|  | Reverse: TCCCAAAGTAGACCTGCCCAGAC |
| IFN-γ | Forward: CCAACGCAAAGCAATACATGA |
|  | Reverse: TTTTCGCTTCCCTGTTTTAGCT |
| IL-6 | Forward: AGGGCTCTTCGGCAAATGTA |
|  | Reverse: GAAGGAATGCCCATTAACAACAA |
